# Intralingual Variation in Acceptability Judgments and Production: Three Case Studies in Russian Grammar

**DOI:** 10.3389/fpsyg.2020.00348

**Published:** 2020-03-31

**Authors:** Anastasia Gerasimova, Ekaterina Lyutikova

**Affiliations:** ^1^Department of Theoretical and Applied Linguistics, Lomonosov Moscow State University, Moscow, Russia; ^2^Pushkin State Russian Language Institute, Moscow, Russia

**Keywords:** acceptability judgments, gradience, production, experimental linguistics, variation, Russian

## Abstract

This paper contributes to the task of defining the relationship between the results of production and rating experiments in the context of language variation. We address the following research question: how may the grammatical options available to a single speaker be distributed in the two domains of production and perception? We argue that previous studies comparing acceptability judgments and frequencies of occurrence suffer from significant limitations. We approach the correspondence of production and perception data by adopting an experimental design different from those used in previous research: (i) instead of using a corpus we use production data obtained experimentally from respondents who are later asked to make judgments, (ii) instead of pairwise phenomena we examine language variation, (iii) judgments are collected formally using the conditions and materials from the production experiment, (iv) we analyze the behavior of each participant across the production and acceptability judgment experiments. In particular, we examine three phenomena of variation in Russian: case variation in nominalizations, gender mismatch, and case variation in paucal constructions. Our results show that there is substantial alignment between acceptability ratings and frequency of occurrence. However, the distribution of frequencies and acceptability scores do not always correlate. Speakers are not consistent in choosing a single variant across the two types of experiment. Importantly, the types of inconsistency they display differ, which means that the variation can be characterized from this point of view. We conclude that the degree of coherence of the two experiments reflects the effects of the evolution of variation over time. Another result is that elicited production and acceptability judgments vary with respect to how they reveal variation in language. In the case of the development or disappearance of variants, production indicates this earlier than judgments, and the rating task has the effect of restricting the choices available to respondents. However, the production method should not thereby be considered more sensitive. We argue that only a combination of production and judgment data makes it possible to estimate the directionality of changes in variability and to see the full distribution of different variants.

## Introduction

The idea that multiple sources of linguistic evidence provide complementary data is not novel. However, it still remains undetermined how different corpus and behavioral measures relate to each other. In this paper, we explore the correlation between the two linguistic domains of production and perception, by assessing the alignment between elicited production and acceptability judgments in the context of language variation.

Traditionally, acceptability judgments have served as the primary source of data for investigators engaged in developing linguistic theories. As the gathering of judgments has become more advanced (see Schütze, 1996; Featherston, 2007; Sprouse, 2007; among others) researchers have begun to use complex non-binary scales, such as the Likert scale. Consequently, the issue of the interpretation of gradience in judgment data has become more prominent. Although judgments are known to be gradient, it is not clear where this gradience comes from Phillips (2009), Schütze and Sprouse (2013), and Sprouse (2015). On one hand, gradience may result from factors other than grammar that affect language processing and decisions about acceptability, e.g., parser limitations and high working memory costs. Another option is that grammatical knowledge is itself gradient: combinations of different grammatical constraints lead to a range of grammaticality^1^ levels.

Our assumptions about the grammatical architecture restrict our predictions with respect to different data sources. If grammar is considered categorical, gradience is reduced to an effect of extra-grammatical factors, i.e., processing mechanisms, which might differ in production and perception. Meanwhile, if grammar is gradient, we expect consistency in the data regardless of the source, be it judgments or produced texts. Consequently, the level of correspondence observed between the two language domains might shed light on what type of language modeling is preferable.

Our paper contributes to defining the relationship between production and perception by comparing the results of production and rating experiments in the context of language variation. We find two main problems with previous research on comparison between data sources. The first is that the production data used was retrieved from corpora. This approach has a serious drawback in that a particular selection of texts might not be comparable to the idiolects of the respondents giving their judgments. The second limitation is that the research was primarily focused on pairwise phenomena. This posits a conflict in terms of the dimensions of the data: while we expect a gradient scale of acceptability, we assume a binary choice in production. In this paper, we aim to provide a solution to both of these problems by analyzing the distribution of grammatical options in both the production and perception domains of individual speakers. In particular, we obtain both production and judgment data experimentally, using the same experimental conditions. Moreover, we examine three phenomena of variation in Russian, of the following type: variants are expected to exhibit different levels of acceptability, but none of them are prohibited in any particular context. Finally, we analyze the behavior of each participant individually, which helps us to understand the objective laws behind the data correspondence.

The rest of the paper is organized as follows. In section “Theoretical Background,” we provide a brief overview of previous work on comparison between data sources, which includes the results of linking acceptability ratings with corpus data and other experimental methods. In section “The Present Study,” we discuss the implicit assumptions behind the hypotheses tested in the previous research and formulate the objectives of the present study. This section also presents the materials for the experimental study – three types of constructions in Russian that display a certain degree of variability. In section “Experiments,” we provide a description of the two series of experiments, involving production and judgments, conducted on the same sample of participants. In the same section, we estimate the level of correspondence between the two types of experiments by checking respondents’ individual results. Section “Discussion of the Experimental Results” discusses the theoretical consequences of our findings. Final section concludes the study.

## Theoretical Background

### Linking Acceptability Ratings and Corpus Data

Several recent studies investigate the relationship between acceptability judgments and frequency of occurrence. The main hypothesis is that grammatical knowledge is probabilistic and determines both frequency of occurrence and acceptability ratings. Consequently, on the basis of probabilities found in a corpus, one ought to be able to predict acceptability judgments. To formalize the gathering of these probabilities, investigators used language models that were fitted to the annotated corpus data in a supervised (Bresnan, 2007) or unsupervised manner (Lau et al., 2017; Sprouse et al., 2018).

Bresnan (2007) explored the correspondence between the two data sources with respect to the English dative alternation (e.g., *give the boy the book* vs. *give the book to the boy*). Using several contextual predictors, including various properties of the recipient and the theme, in the Switchboard corpus of spontaneous speech, the researcher created a statistical model that successfully predicted the choice of dative construction on the annotated test set. Then two experiments were conducted, which evaluated how the ratings provided by speakers correspond to the probabilities predicted by the model. The results showed that acceptability judgments corresponded to corpus probabilities. Even more importantly, linguistic manipulations with contextual predictors affected both probabilities and acceptability judgments in the same direction.

A conceptually different approach was proposed by Lau et al. (2014, 2015, 2017). In this study, acceptability judgments were predicted by unsupervised language models trained on raw text which did not contain any annotation or set predictors (in contrast with Bresnan, 2007). As likelihood of occurrence is partially determined by sentence length and lexical frequency, probabilistic language models were augmented with acceptability measures that compensate for additional frequency factors. The language models were tested on a dataset that contained sentences at varying levels of acceptability: original sentences retrieved from the British National Corpus and mappings of these sentences with errors introduced by round-trip machine translation^2^. Lau et al. (2017) then computed the Pearson correlation coefficient between the acceptability scores produced by computer models and mean human judgments. The comparison showed that some models achieved good levels of accuracy in predicting the observed gradient data. This result recommends these models as more effective than traditional formal grammars, which are unable to predict acceptability gradience at all.

A replication of this study was performed by Sprouse et al. (2018). The major criticism of the results from Lau et al. (2014, 2015, 2017) concerns the fact that round-trip translations might not create grammatical oppositions of the kind usually devised by syntacticians, whereby a specific grammatical property is manipulated while other properties remain constant in an experimental set. To formalize comparison between classical formal grammar and probabilistic language models with respect to accommodating gradient data, the datasets were enriched by randomly selected samples of pairwise and multi-condition phenomena. The results show that probabilistic models demonstrate a substantial loss in coverage of phenomena that are captured by categorical grammars and can be revealed in controlled syntactic experiments. In particular, the models fail to capture up to 35% of the phenomena that are accounted for in modern generative theory.

Notably, the three studies just mentioned relate acceptability judgments to production data retrieved from a corpus. This presupposes that the corpus embodies the grammatical constraints that are implied by speakers in rating tasks because all the text entries were produced by speakers of the same language. However, this approach has its limitations. Whether corpora correctly capture usage is still an open question. It is also essential to consider what types of texts are represented in corpora. While Bresnan (2007) used the Switchboard corpus of spontaneous speech, in the study by Lau et al. (2017) this factor was not controlled for, and Sprouse et al. (2018) used utterances from research literature. The problem is that data from texts belonging to particular genres might not be comparable to the results of acceptability tasks in which speakers are asked to evaluate the *naturalness* of the stimuli.

Another drawback concerns the type of data used in a language model: predictors identified by linguists, or features yielded in an unsupervised manner. Where a researcher uses predictors, it is doubtful whether all the predictors affecting the final result are in fact being distinguished. Additionally, it is unclear how to interpret findings at the lower end of the frequency spectrum. Testing on the basis of predictors is subject to limitations, as the corpus might lack all the possible combinations of predictor values that would be required by a comprehensive test. This problem was addressed by Divjak (2017), who analyzed *that*-clauses in Polish and encountered difficulties in determining which variables had an impact on acceptability ratings. Divjak (2017) suggests that implicit probabilistic syntactic knowledge is based not on n-gram frequency, but rather on higher-order knowledge (involving schemata or rules). However, the lack of any clear correspondence between frequency and ratings could result from the low capacity of the corpus.

The use of unsupervised language models is not trouble-free either. Language models take into account all kinds of information that can be retrieved from a corpus, which is not necessarily the same information that humans obtain when they acquire and use language. Thus, the replacement of the existing theoretical grammar models with computational ones would eliminate the explanatory function of language theory and modeling.

Taken together, the examined studies point toward the problem of corpus representativeness, which leads to flaws in the comparison between usage data and acceptability ratings. A possible solution would be to limit production data to the phenomenon under observation and obtain it specifically for the comparison at issue. In the following section, we review existing studies that have used a different source for production data, and provide the rationale for the present work.

### Gathering Production Data Differently

A group of studies have approached the question of the correspondence between production and perception data by adding rigor to the production data gathering process. Instead of using language models trained on large datasets, the researchers obtained production frequencies in experiments.

To our knowledge, the first attempt to connect acceptability ratings to experimentally obtained production data was made by Adli (2011), who investigated the preferred subject position in Spanish *wh*-questions with respect to the thematic role of the *wh*-word. The database of elicited speech turned out to be rather limited: one expected option was completely absent. Hence, the representativeness of the database limited the potential for meaningful comparison.

The next study was carried out by Verhoeven and Temme (2017), who used a forced-choice task to evaluate results of production. They investigated the choice between SO and OS order in German clauses using two experimental procedures: forced-choice and split-100 rating. It was assumed that at some point in the production process, the speaker would compare a set of alternative expressions and judge their relative appropriateness in a particular context. This assumption is questionable as there is evidence that forced-choice is a form of rating task. Sprouse et al. (2018) reports that the results of forced-choice tasks, when transformed into ratings by means of the Elo system first developed for rating the relative strength of chess players, in fact correspond directly to the results of Likert scale tasks. The results of the experiments by Verhoeven and Temme (2017) turned out to be highly correlated. We think this is presumably due to the fact that speakers were ultimately carrying out the same rating task in both experiments^3^.

Another attempt to relate acceptability ratings and elicited usage data was by Bermel et al. (2018), who retrieved probabilities from a balanced corpus and compared them to the distribution of existing options in fill-in-the-gap and rating tasks, completed by respondents simultaneously. Bermel et al. (2018) took the responses to the fill-in-the-gap task to serve as production data; however, they observed that this could more accurately be thought of as a forced-choice situation, as there were only two possible options in the two syntactic contexts. Although a correlation was found, there is a limitation to this study, namely, the performance of two distinct tasks within a single questionnaire. The main drawback of such a procedure is that the acceptability ratings could influence the production results and vice versa.

To summarize this brief review, we argue that previous comparisons of acceptability judgments and production based on the information retrieved from corpora have the following limitations. First, the corpus data may incorrectly represent the speech of the respondents providing judgments, due to differences in the text types involved. Second, speech corpora give rise to difficulties in dealing with low frequency spectrum phenomena. Third, the use of probabilistic language models raises the issue of model parameters. Where predictors have been pre-defined by linguists it is unclear whether the whole range of predictors affecting the final result has been taken into consideration. In the case of unsupervised feature detection, the algorithm may use all kinds of information that can be retrieved from the corpus, which is not necessarily the information that humans obtain when they acquire and use language. Finally, those studies which aimed to control for relevant factors when gathering production data did not change the overall picture. Intrinsically, these studies were comparing different acceptability rating methods and considering how well their results correspond to the predictions of probabilistic language models. In the next section we suggest how the research question can be modified to overcome these limitations.

## The Present Study

Before we formulate the objectives of the present study, we would like to discuss the implicit assumptions behind the hypotheses tested in the previous research. The fundamental idea concerns the nature of grammatical knowledge: if grammar is probabilistic, it determines both offline production and comprehension, which are externalized quantitatively in frequency of occurrence and acceptability ratings, respectively. However, it is essential to ascertain what kind of grammatical knowledge is presupposed in this approach. In most of the studies discussed above, production data was retrieved from a corpus and was compared to acceptability ratings provided by a group of speakers. That is, the relevant instances of production were determined by the grammatical knowledge of the individuals who produced the set of texts that happened to be included in the corpus. Production data in this case reflects ‘collective grammar,’ which is not necessarily reducible to a simple sum of individual idiolects (Bailey, 1973; Bickerton, 1975; Wolfram and Beckett, 2000; Kuhl, 2003) and represents the individual grammars only to the extent of what is present in the texts. Meanwhile, judgment data is determined by the grammatical knowledge of speakers who participate in the survey, representing another form of ‘collective grammar.’ The question therefore arises as to whether investigators are comparing entities of the same nature when looking for correlations between frequencies and ratings.

In general, it is presupposed that an individual belonging to a language community possesses the same grammar as the people with whom she communicates – that is, other members of the same community or social group (Horvath and Sankoff, 1987). This methodology is based on the “homogeneity assumption” that individual-speaker variation is not important in describing variation in general (Wolfram and Beckett, 2000). In the reviewed research a conceptually similar idea is assumed, namely that those speakers who participate in the surveys possess the same grammar as those who composed the texts found in the corpus. However, this assumption is untenable because it is possible that the language community providing the production frequencies and the individuals providing the ratings possess grammars that are far from equal. In other words, using a corpus means that an additional factor needs to be taken into account: the level of coherence between the grammar of the survey participants and the collective grammar reflected in the corpus.

We suppose that if grammatical knowledge is indeed probabilistic, one would see consistent patterns in the production and comprehension of a single speaker, without the mediation of the collective language system of all speakers or speakers from a certain community. Our prediction is that in this case there would be a one-to-one correspondence between the production data and acceptability judgments of a given speaker. Both production and judgment data should provide the same ranking of variants: the most frequent variant would also be the most acceptable, and the least frequent variant would be the least acceptable.

Another important issue is connected to the type of phenomena on the basis of which the two language domains were compared. In most of the studies mentioned, linguists analyzed alternations that were dependent on a set of contextual predictors, distinguished and annotated by investigators in advance. This means that there were contexts where one alternative was acceptable while the other was not. Although the question regarding the completeness of the set of predictors remains open, the distribution of predictors might dictate the quantitative values for frequency of variants. Consequently, the ratings for a certain phenomenon are not directly compared to the distribution of that phenomenon but instead to the distribution of predictors that favor a certain value. The relation between the distribution of predictors and the distribution of variants might be non-linear, at least because not all the predictors are distinguished and there may be interaction between them. We do not aim to explore the nature of this relationship; our point is that such an approach lends additional complexity to any hypothesis about the relation between offline perception and production.

The analysis of pairwise phenomena, as in Lau et al. (2017) and Sprouse et al. (2018), is also insufficient. It presupposes a binary distribution of language data: (i) without any violations of functional/grammatical constraints, (ii) with such violations. In this case, the comparison is carried out between variables of different dimensions: in production there is a binary choice, between producing and not producing a construction, while in perception there is a scale of acceptability.

To avoid the issue of predictors and problems arising from the binary distribution of language data, we suggest studying phenomena that supposedly exhibit free variation: although variants may favor certain contexts, none of them seem to violate any constraint and thus to be unacceptable in any particular context.

Therefore, in the present study, we use a hypothesis on the correspondence between offline production and comprehension that requires fewer assumptions than the hypotheses used in previous research. We address the following research question: how are the grammatical options distributed in both the production and perception domains of a single speaker? We believe that answering this question will contribute to the task of connecting gradient acceptability judgments and usage, as it eliminates the problems of corpus representativeness and binary opposition in the language phenomena under examination.

We approach the correspondence of production and perception data by adopting an experimental design alternative to those used in previous research. Firstly, instead of using a corpus we use production data obtained experimentally from respondents who are later asked to make judgments. Secondly, instead of pairwise phenomena we examine language variation. The phenomena that we explore include those involving more than two alternatives, to the effect that we do not end up with a forced-choice task when gathering production data. Thirdly, judgments are collected formally using the conditions and materials from the production experiment. Finally, we analyze the behavior of each participant across the production and acceptability judgment experiments.

### The Phenomena Under Observation

We examine three phenomena of variation in Russian. The choice of phenomena was premised on the status of variation: we aimed to use both data with predictors and data with free variation. This way we could replicate the choice of data from both types of study undertaken previously: those that used data with annotated predictors, and those that used raw data^4^.

The first phenomenon addressed is **case variation in nominalizations.** Russian event nominalizations belong to the ergative-possessive type (Koptjevskaja-Tamm, 1993), which means that arguments of intransitives and internal arguments of transitive stems are marked with the possessive, genitive case (GEN), while external arguments of transitives are assigned instrumental case (INSTR). However, for some stems the external argument can be marked both GEN and INSTR: this is possible for nominalizations with a lexically governed internal argument (1) and for nominalizations derived from unergative stems (2). That is, the case marking strategy is one of the parameters of intralingual variation for Russian.


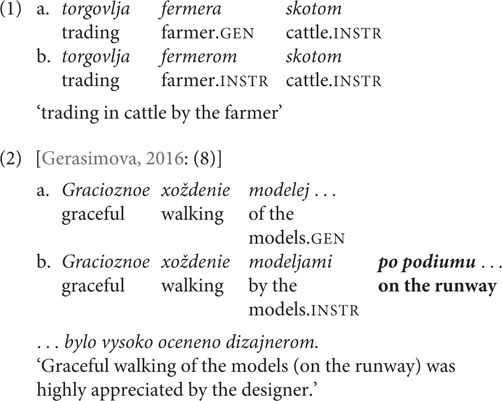


The case marking strategy depends on the structural properties of the nominalization: thus, adverbial PP modification increases the acceptability of INSTR (2) (Pereltsvaig, 2017), an observation supported by the experimental data from Pereltsvaig et al. (2018). This aspect is modeled within the framework of formal syntax in terms of the amount of structure that is nominalized: the syntactic structure is claimed to be more complex when an adverbial PP is merged, which makes it similar to the structure of transitives. Pereltsvaig (2017) connects the larger structure of nominalization with the licensing of INSTR. This means that even when there is no adverbial PP modification of a nominalization with a lexically governed internal argument, but its external argument is nonetheless marked INSTR, the nominalization is supposed to possess a larger structure. If we rely on the theoretical modeling proposed by Pereltsvaig, we might suppose that in the absence of a PP the smaller structure would be preferred on the basis of Economy Principle considerations. Therefore, a general preference for GEN is expected for both production and acceptability judgments. With respect to our goals, this phenomenon presents variation with binary choice and no identified predictors.

The second phenomenon is **gender mismatch**, which occurs in the context of masculine nouns that denote a professional status and refer to females. These nouns can trigger both masculine and feminine agreement on attributive modifiers and past tense verbs (Muchnik, 1971; Crockett, 1976; Shvedova, 1980; Pesetsky, 2013; Lyutikova, 2015; among others). The three possible agreement patterns are: GRAMMATICAL AGREEMENT, where all agreeing constituents are masculine (3a), REFERENTIAL AGREEMENT, within which modifiers are masculine and the verb is feminine (3b), and REFERENTIAL ATTRIBUTIVE AGREEMENT, where non-classifying adjectives [adjectives without an idiomatic interpretation (Rothstein, 1980; Svenonius, 2008; Pesetsky, 2013)] and the verb are feminine (3c). The majority of investigators suggest that the observed variation results from a process of “feminization” at some stage in the derivation, which henceforth determines the agreement pattern of the nominal (Pereltsvaig, 2006, 2015; Asarina, 2009; Pesetsky, 2013; Lyutikova, 2015; Puškar, 2017; Steriopolo, 2018; and others). To date, no specific factors have been identified as influencing the choice of agreement pattern. REFERENTIAL AGREEMENT is assumed to be the most frequent pattern in actual usage: consequently, we would expect it to be the most used and the most acceptable pattern in the experiments. This variation presents multiple agreement choices, not limited to binary distribution: the three mentioned patterns are all considered acceptable by both traditional grammars and formal syntactic studies.


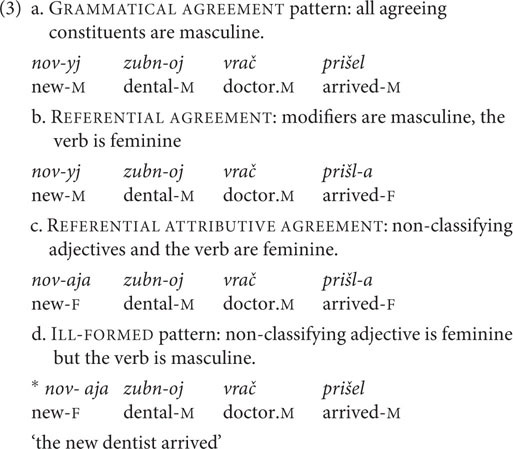


The third phenomenon is **case mismatch in paucal constructions.** In paucal constructions feminine nominalized adjectives and adjectives that modify feminine nouns can be marked either NOM or GEN (4)–(5) (Graudina et al., 1976; Shvedova, 1980; Golub, 1997; and others). The choice of case marking partially depends on the context of the paucal construction: NOM is preferred in argumental (DP) position, where the paucal construction agrees with the predicate, and GEN is used primarily in quantificational (QP and PP) positions, where there is no predicate agreement (Shkapa, 2011; Lyutikova, 2015). Corpus studies by Shkapa (2011) and Gerasimova (2019) have shown that in general the NOM form is more frequent in paucal constructions. Therefore, NOM is expected to be the preferred option in both production and perception experiments. Some previous studies also claim that the choice of case marking on the adjectival constituent depends on internal properties of the paucal construction, such as the morphological type of the adjective or stress position on the noun. However, according to Shkapa (2011) there is no evidence for these predictions. We consider this variation to have an identified predictor, namely, the presence of predicate agreement.


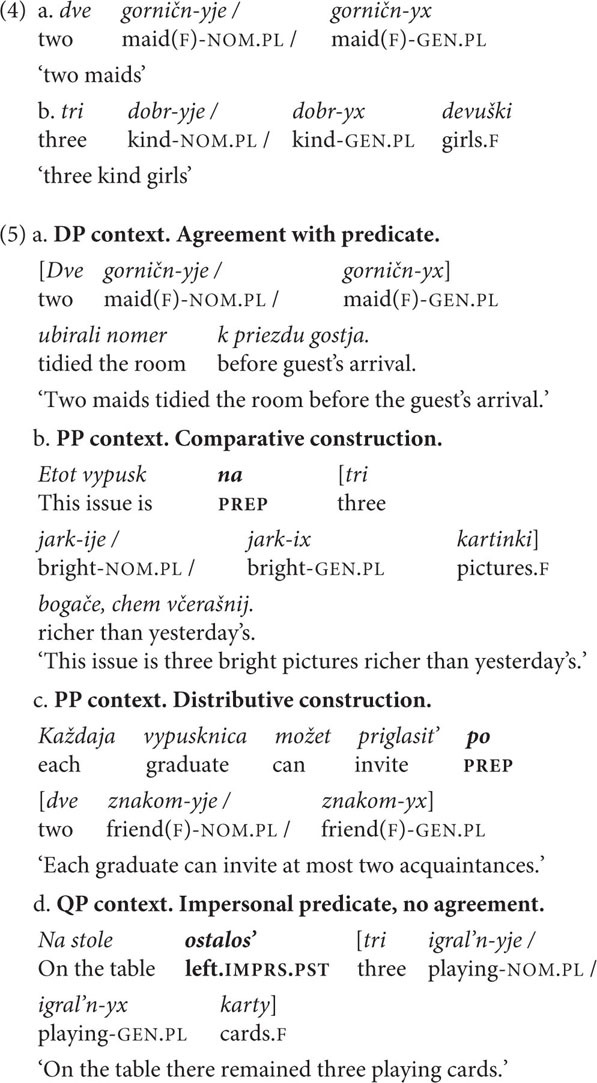


To sum up, we have chosen three phenomena that differ with respect to the type of variation they display. Firstly, in all three cases two or more variants are acceptable and none of the variants explicitly violates any functional or grammatical constraints. Nonetheless, there are some predictions with respect to the most frequent option (case or agreement pattern). Secondly, the variation is not fully determined by predictors. Only in the case of paucal constructions are contextual predictors identified, although their presence does not guarantee any particular choice (as shown in Shkapa, 2011). In the case of nominalizations, variation can be manipulated by adding an adverbial PP into the structure; when there is no PP the variation is considered to be free. It is not known how gender mismatch can be manipulated either. Finally, it may be that the variants are distributed unequally over speakers. In particular, in Pereltsvaig et al. (2018) it was shown that some speakers are consistent in using both GEN and INSTR, while some do not allow INSTR at all. There is no similar data for gender mismatch and paucal constructions; however, it is possible that these two phenomena are also characterized by a cross-speaker distribution of variants. This property of the variation should not influence the hypothesis testing, as in case there is any intraspeaker variation we would expect a speaker to be consistent in her choices in both perception and production.

The reviewer raised the issue of the linguistic comparability of the phenomena with respect to their source. The three phenomena under discussion appear to be grammatically comparable due to the uniformity of the syntactic structures and mechanisms behind feature interpretation and valuation within a given language (Adger and Svenonius, 2011)^5^. All three involve variation that arises in the process of feature valuation with respect to the constituent that enters derivation bearing an unvalued feature. Variation results from the fact that there is more than one controller available for feature valuation: the gender agreement controller in case of gender mismatch, and the case governor for nominalizations and paucal constructions. The availability of multiple controllers may be inherent to the structure (as in paucal constructions) or originate from conscious or subconscious structure varying (as in the case of gender mismatch and nominalizations, respectively). On the basis of these observations we suppose that the three investigated phenomena can be attributed to the same component of grammar, namely, narrow syntax, and can be assumed to involve the same type of grammatical operation, viz., feature valuation.

## Experiments

In order to investigate the correspondence between the distribution of grammatical options in both offline production and offline perception of a single speaker we conducted a series of linguistic experiments using the three Russian phenomena presented above. For each phenomenon, we carried out two experiments: a production experiment, in which respondents were asked to provide the case/agreement morphology themselves, and an acceptability judgment experiment, in which respondents provided acceptability judgments using a 5-point Likert scale. We first conducted the three production experiments, one for each phenomenon; then 5 months later the three judgment experiments were launched. In both sets of experiments, we made use of the same group of participants. We suppose that the chosen period between the sets of experiments was long enough to eliminate any syntactic satiation effect. In addition, as we were using the same materials in both it was necessary that the speakers forget the stimuli in the intervening period. We assume that a span of several months is sufficient to achieve both goals: however, there is more to be done with respect to defining the proper timing for such a series of experiments^6^. When participating in a set of experiments, respondents completed separate experiments in one day with breaks half an hour long in between: the respondent first completed the nominalization experiment, then the gender mismatch experiment, and finally the experiment on paucal constructions. All participants encountered the experiments in the same order. The breaks between experiments were arranged in order to avoid fatigue effects.

### Participants

One hundred and ten self-reported native Russian speakers participated in the three production experiments (82 females). Ages ranged from 15 to 49 (mean age 21, SD 5.3). Fifty-eight of these participants subsequently completed the three acceptability judgment surveys (43 females). This time ages ranged from 17 to 37 (mean age 21, SD 4.7). All participants provided informed consent and were naïve as to the purpose of the study and the research question. The experiments were carried out in accordance with the Declaration of Helsinki and the existing international regulations concerning ethics in research. The participants performed the task remotely, via the web-based software Google Forms. Participants were presented with one sentence at a time; the time allowed for the answer was not limited but participants were instructed to complete the task as fast as possible.

### Materials and Procedure

In this section, we discuss experimental materials for each phenomenon. We first review the experimental factors and the number of stimuli in both production and acceptability judgment experiments. Then, we describe the sample stimuli and the production task. In all production experiments, the task for respondents was to provide the case or agreement morphology, and the only differences concern how the material to be filled in was presented. The section ends with a discussion of the item-to-filler ratio, the training sentences and the procedure involved in the acceptability judgment experiments. In all the experiments reported in this study counterbalancing was achieved by means of pseudorandomization and a Latin square design.

In the case of **nominalizations**, there was only one factor in the production experiment – the type of nominalized verbal stem. These are transitive stems with lexically governed internal argument and unergatives, for which we expected variation, versus ‘normal’ transitives and unaccusatives, for which we expected no variation and that were used as baseline conditions. We constructed 16 target sentences, four for each of the four conditions. The target sentences were presented in four pseudorandomized orders and interspersed with 32 filler items of comparable structure and length, which contained participles instead of nominalizations.

In each condition from the production experiment, there was a choice between GEN and INSTR. Therefore, in the acceptability judgment experiment one more factor was added, namely, the case marking of the external argument. The number of stimuli from the production experiment was multiplied by two, giving 32 sets of target sentences in the judgment experiment. We used the 16 sets of stimuli that had already been used and added 16 more sets (see Supplementary Data Sheet 1 for production experiment stimuli). Sample stimuli from the Table 1 represent one set. The number of filler sentences was kept the same in order to avoid fatigue effects.

**TABLE 1:** Conditions from the nominalization experiments.

Condition	Type of nominalized stem	Case of external argument (judgment experiment only)	Example
1–2	Transitive	GEN-INSTR	V tot mesjac **armija** osvobodila **stolitcu**, i osvoboždenie **armii/armiej** stolicy sil’no podnjalo boevoj dux soldat.
			That month **army.NOM** reconquered **capital.ACC**, and reconquest **army.GEN/army.INSTR capital.GEN** greatly lifted the martial spirit of the soldiers.
3–4	Transitive with lexically governed internal argument	GEN-INSTR	V techenie matča **sud’ja** podygryval **komande**, a podygryvanie **sud’i/sud’ej komande** strogo zapreščeno po pravilam čempionata.
			During the game **referee.NOM** favored **team.DAT,** and favoring **referee.GEN/referee.INSTR team.DAT** is strictly prohibited by the championship rules.
5–6	Unergative	GEN-INSTR	Posle procedury **pacient** stal kašljat’, i kašljanie **pacienta**/**pacientom** srazu nastorožilo lečaščego vrača.
			After the procedure **patient.NOM** began to cough, and coughing **patient.GEN/patient.INSTR** immediately attracted the doctor’s attention.
7–8	Unaccusative	GEN-INSTR	Každuju osen’ **babuška** priezžala k nam v gorod, i priezd **babuški/babuškoi** vsegda soprovoždalsja vkusnym i sytnym zastol’em.
			Every autumn **grandmother.NOM** arrived in the city, and arrival **grandmother.GEN/grandmother.INSTR** was always followed by a holiday feast.

Each stimulus was constructed in the following manner: the first part of the sentence contained the finite verb with its arguments, and the second part contained the nominalization formed from that verb. In the production experiment, speakers were asked to generate arguments of nominalizations, assigning the case that sounded most natural to them in each instance. The second conjunct of a complex sentence contained a gap which the participant had to fill in with the argument from the preceding context (the first conjunct of the sentence) (6).


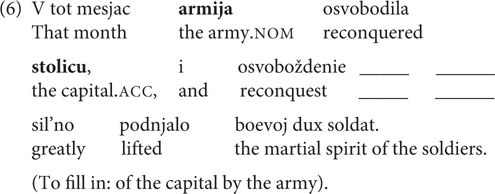


In the **gender mismatch** experiment, we examined gender agreement for various combinations of adnominals (determiners: possessive and demonstrative pronouns; high adjectives; low adjectives). All eight combinations that were used are listed in (7).


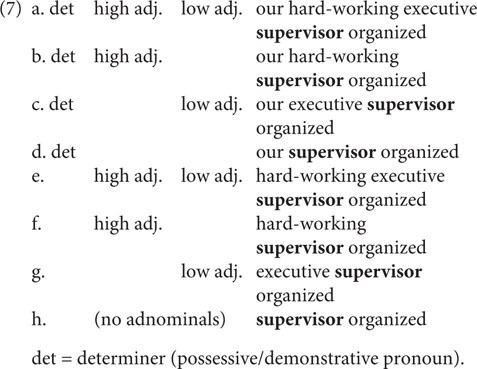


Each combination from (7) was used twice in the experiment, which yields 16 sets of experimental sentences (see the sample stimuli in Table 2). Thirty-two filler items contained nouns that unambiguously denote the sex of the referent.

**TABLE 2:** Conditions from the gender mismatch experiments.

Condition	Adnominals in NP	Agreement pattern (judgment experiment only)	Example
1	Det High Low	GRAMMATICAL	Vsju noch’ Tane ne udalos’ somknut’ glaz: **nash otvetstvennyj proektnyj** menedzher **gotovil** prezentaciju reklamnoj kampanii dlja radioholdinga.
			Tanja couldn’t get a wink of sleep all night: **our.M responsible.M project.M** manager **was preparing.M** the presentation of a promotional campaign for the radio corporation.
2		REFERENTIAL	**our.M responsible.M project.M** manager **was preparing.F**
3a		REFERENTIAL ATTRIBUTIVE	**our.F responsible.M project.M** manager **was preparing.F**
3b		REFERENTIAL ATTRIBUTIVE	**our.F responsible.F project.M** manager **was preparing.F**
4		ILL-FORMED	**our.M responsible.F project.M** manager **was preparing.M**
5	Det High	GRAMMATICAL	**our.M responsible.M** manager **was preparing.M**
6		REFERENTIAL	**our.M responsible.M** manager **was preparing.F**
7a		REFERENTIAL ATTRIBUTIVE	**our.F responsible.M** manager **was preparing.F**
7b		REFERENTIAL ATTRIBUTIVE	**our.F responsible.F** manager **was preparing.F**
8		ILL-FORMED	**our.M responsible.F** manager **was preparing.M**
9	Det Low	GRAMMATICAL	**our.M project.M** manager **was preparing.M**
10		REFERENTIAL	**our.M project.M** manager **was preparing.F**
11		REFERENTIAL ATTRIBUTIVE	**our.F project.M** manager **was preparing.F**
12		ILL-FORMED	**our.M project.F** manager **was preparing.M**
13	Det	GRAMMATICAL	**our.M** manager **was preparing.M**
14		REFERENTIAL	**our.M** manager **was preparing.F**
15		REFERENTIAL ATTRIBUTIVE	**our.F** manager **was preparing.F**
16		ILL-FORMED	**our.F** manager **was preparing.M**
17	High Low	GRAMMATICAL	**responsible.M project.M** manager **was preparing.M**
18		REFERENTIAL	**responsible.M project.M** manager **was preparing.F**
19		REFERENTIAL ATTRIBUTIVE	**responsible.F project.M** manager **was preparing.F**
20		ILL-FORMED	**responsible.M project.F** manager **was preparing.M**
21	High	GRAMMATICAL	**responsible.M** manager **was preparing.M**
22		REFERENTIAL	**responsible.M** manager **was preparing.F**
23		REFERENTIAL ATTRIBUTIVE	**responsible.F** manager **was preparing.F**
24		ILL-FORMED	**responsible.F** manager **was preparing.M**
25	Low	GRAMMATICAL	**project.M** manager **was preparing.M**
26		REFERENTIAL	**project.M** manager **was preparing.F**
27		REFERENTIAL ATTRIBUTIVE	**project.F** manager **was preparing.F**
28		ILL-FORMED	**project.F** manager **was preparing.M**
29	No	GRAMMATICAL	manager **was preparing.M**
30		REFERENTIAL	manager **was preparing.F**

In the judgment experiment, four patterns were examined for each combination: GRAMMATICAL AGREEMENT, REFERENTIAL ATTRIBUTIVE AGREEMENT, REFERENTIAL AGREEMENT, and ILL-FORMED AGREEMENT patterns. Two important properties of the stimuli must be pointed out. Firstly, for combination (7h) only two agreement patterns were logically available (GRAMMATICAL AGREEMENT and REFERENTIAL AGREEMENT). As shown in Table 2, conditions 29 and 30 correspond to this combination. Secondly, for combinations (7a) and (7b) the REFERENTIAL ATTRIBUTIVE AGREEMENT pattern could be applied in two ways: either only the determiner demonstrates feminine agreement and the high adjective remains masculine, or both determiner and high adjective are feminine. Pesetsky (2013) considers both variants to be equally acceptable; in contrast, Pereltsvaig (2015) predicts that the two adnominals cannot be mismatched. As there is no agreement between investigators and no experimental data that would provide evidence for either point of view, we introduced the two possibilities as two separate conditions: conditions 3a and 3b, 7a and 7b in Table 2 for combinations (7a) and (7b), respectively. Consequently, the two factors, combination and agreement pattern, adjusted according to the considerations mentioned above give 32 conditions in total. In each experiment, there were two sentences for each condition. Fillers were the same as in the production experiment. We chose these quantities of target and filler items in order to avoid fatigue effects.

The target items were complex sentences, in which the first clause provided a context that explicitly indicated the gender of the human denoted by the subject in the second coordinate clause. This was done by using traditionally female names. This part of the sentence involved no agreement morphology. The second clause contained a noun phrase and a verb in the past tense, with gaps instead of endings in the production experiment. Speakers were asked to write the attributive modifiers and the verb with the endings in the textbox so that the sentence was complete (8).


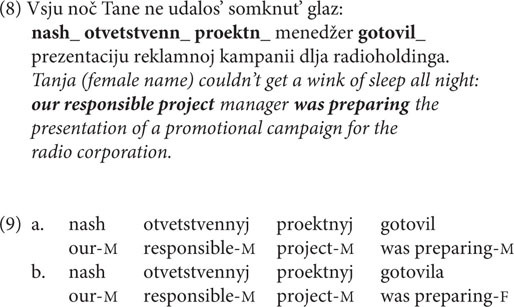


In the **paucal constructions** production experiment, we controlled for context (QP, DP, and PP), animacy, and pattern, i.e., whether the paucal construction involved feminine nominalized adjectives or modified feminine nouns. This gives 12 conditions in total. With two sentences for each condition there were 24 sets of target sentences. The sentences were kept relatively short. The target sentences were interspersed with 48 filler items of comparable structure and length, which contained numeral constructions involving other numerals and nouns of different grammatical genders.

The acceptability judgment experiment involved one more factor – case: in each condition from the production experiment there was a choice between NOM and GEN. Therefore, the number of stimuli in the judgment experiment was multiplied by two in comparison to the production experiment (see Table 3 for the sample set of stimuli). Filler items were kept the same.

**TABLE 3:** Conditions from the paucal construction experiments.

Condition	Context	Pattern	Animacy	Case	Example
1–2	DP	Nominalized adjective	Animate	NOM-GEN	Dve **beremennye/beremennyx** obsuždali novosti sidja na skamejke. Two **pregnant woman(F)-NOM.PL/pregnant woman(F)-GEN.PL** were discussing the news sitting on a bench.
3–4	DP	Nominalized adjective	Inanimate	NOM-GEN	Dve **pračečnyje/pračečnyx** byli otremontirovany v gorode v etom mesjace. Two **laundry.NOM.PL/laundry.GEN.PL** have been renovated in the town this month.
5–6	DP	Noun	Animate	NOM-GEN	Tri **veselye/veselyx devočki** obsuždali plany na vyxodnye. Three **cheerful-NOM.PL/cheerful-GEN.PL girls** were discussing plans for the weekend.
7–8	DP	Noun	Inanimate	NOM-GEN	Dve **sočnye/sčnyx gruši** byli ostavleny v novoj vaze. Two **juicy-NOM.PL/juicy-GEN.PL pears** were left in a new bowl.
9–10	QP	Nominalized adjective	Animate	NOM-GEN	Včera za etot srok prinjato dve **beremennye/beremennyx**. Yesterday in the same period an appointment was given to two **pregnant woman(F)-NOM.PL/pregnant woman (F)-GEN.PL**.
11–12	QP	Nominalized adjective	Inanimate	NOM-GEN	V etom rajone za god obustroeno **dve pračečnye/pračechnyx.** In this neighborhood within a year there were equipped two **laundry.NOM.PL/laundry.GEN.PL.**
13–14	QP	Noun	Animate	NOM-GEN	V sledujuščii etap viktoriny prošlo dve **veselye/veselyx devočki.** Into the next stage of the quiz were accepted three **cheerful-NOM.PL/cheerful-GEN.PL girls.**
15–16	QP	Noun	Inanimate	NOM-GEN	Na stole k večeru ostalos’ dve **sočnye/sočnyx gruši.** On the table by the end of the day there remained two **juicy-NOM.PL/juicy-GEN.PL pears**.
17–18	PP	Nominalized adjective	Animate	NOM-GEN	Za každyi čas vrač prinimaet po dve **beremennye/beremennyx.** Every hour the doctor gives an appointment to two **pregnant woman(F)-NOM.PL/pregnant woman (F)-GEN.PL**.
19–20	PP	Nominalized adjective	Inanimate	NOM-GEN	V každom rajone kompanija otkryla po dve **pračečnye/pračečnyx.** In every neighborhood the company opened two **laundry.NOM.PL/laundry.GEN.PL.**
21–22	PP	Noun	Animate	NOM-GEN	Na každuju lavočku režisser posadil po tri **veselye/veselyx devočki.** On every bench the director seated three **cheerful-NOM.PL/cheerful-GEN.PL girls.**
23–24	PP	Noun	Inanimate	NOM-GEN	Každomu gost’u xozjajka vydala po dve **sočnye/sočnyx gruši.** To every guest the hostess gave two **juicy-NOM.PL/juicy-GEN.PL pears**.

In the production experiment, the task was to inflect a paucal construction whose component parts (numeral + noun phrase) were provided in parentheses. The numeral was represented with a digit from 2 to 4, and alongside it there was either a nominalized adjective [as in example (10)], or a noun modified by an adjective, given in the singular. The rationale behind this choice is that in Russian paucal constructions the form of the modifying adjective is plural. The form was given in the singular because otherwise we would have to give the NOM.PL, which might lead respondents to prefer that over the GEN.PL and cause a priming effect.


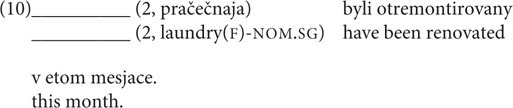


All production tasks were designed so that participants could give only one answer. Only one phenomenon out of three presupposed a binary distribution of answers (namely, nominalizations, where respondents had to choose GEN or INSTR). In the gender mismatch experiment respondents could choose from multiple variants, all of which were restricted to the phenomenon in question, and in the experiment on paucal constructions respondents could choose alternative constructions (the interpretation of digits was not restricted, so respondents could use collective numerals or quantificational nouns; the latter were chosen in 5.33% of responses). The risk we were running with the nominalization experiments was that we would end up with a forced-choice task. However, as was discussed above (see section “Gathering Production Data Differently”), forced-choice should be considered a rating task: therefore, we would not expect any differences in the results between the production and acceptability judgment experiments.

The procedure for all the acceptability judgment experiments was the same. Respondents were asked to rate each sentence on a scale from 1 to 5, where 1 represents *bad* or *unnatural* and 5 represents *good* or *natural^7^*. Participants were told that the task had no correct answers and had nothing to do with what is advocated in prescriptive grammar or the plausibility of the described event.

The first four trials in each experiment served as training sentences and were identical for all participants. Out of the 110 respondents who completed the survey, four participants were excluded as they did not understand the task, yielding 106 participants whose data was later analyzed. As in the production experiments, at the beginning of the judgment experiments there were four training sentences, which provided grounds for excluding any participants who did not provide judgments at the expected end of the spectrum^8^. On the basis of this metric we excluded 1 participant out of the 58 who completed the surveys, which yields 57 participants whose data was later analyzed.

The described quantitative properties of the stimuli from the experiments are presented in Table 4. These numbers and, consequently, the number of stimuli responded to remain the same for all the participants despite the individual results in the production experiments. The number of filler items was adjusted to eliminate fatigue effects: when the number of target sentences was less than 25, the item-to-filler ratio was 1:2, and when there were more the item-to-filler ratio was 1:1. The general principle was not to exceed a total of 100 sentences, giving a survey that could be completed in approximately 15–20 min.

**TABLE 4:** Quantitative properties of stimuli in the experiments.

Experiment	Method	Controlled variables	Number of levels	Conditions	Sentences per condition	Target sets	Fillers
Nominalizations	Production	Type of nominalized stem	4	4	4	16	32
	Judgments	Type of nominalized stem	4	8	4	32	32
		Case of external argument	2				
Gender mismatch	Production	Adnominals in NP	8	8	2	16	32
	Judgments	Adnominals in NP	8	16	2	32	32
		Agreement pattern	2				
Paucal constructions	Production	Context	3	12	2	24	48
		Pattern	2				
		Animacy	2				
	Judgments	Context	3	24	2	48	48
		Pattern	2				
		Animacy	2				
		Case	2				

It is important to note that not all the controlled variables were independent variables, i.e., involved in the hypothesis testing. In particular, the combinations of adnominals from the gender mismatch experiment and animacy in the paucal construction experiment were considered extraneous variables, i.e., they were not intentionally tested in the experiments, but they were controlled for, as there was a possibility that they could influence the final results^9^. There was no effect found for these two variables^10^.

### Data Analysis

All production experiments in the paper were analyzed by means of the same data analysis procedure: each experiment involved from one to three explanatory variables or predictors, and we observed a categorical response with 2 or more values. Therefore, the data from the production experiments were fitted to a logistic regression model (Levshina, 2015) with the following factors: the stem type in the case of nominalizations; number of adnominals in the case of gender mismatch; context, pattern, and animacy in the case of paucal constructions. The model fitting procedure was implemented in R (R Core Development Team, 2015). The goodness-of-fit can be estimated by the concordance index which for the three models was 0.7, which is considered to be acceptable (Hosmer and Lemeshow, 2000).

For all acceptability judgment experiments we also followed the same data analysis procedure. First, the raw judgments were *z-score* transformed in order to eliminate any potential scale bias resulting from differences in how each individual interpreted the scale (Schütze and Sprouse, 2013). All the reported analyses were run on both raw and transformed data; however, there were no differences in the results. In the results reported below, we provide the transformed data. For each experiment, the results of the study were entered in a Repeated Measures ANOVA with acceptability score and {STEM TYPE, CASE} for nominalizations, {ADNOMINALS, AGREEMENT PATTERN} for gender mismatch, {CONTEXT, PATTERN, CASE} for paucal constructions as factors.

### Results

In the case of production, the logistic regression models showed significant effects and interactions of the factors mentioned in 4.4 (*p* < 0.001), except animacy in the paucal construction experiment and adnominal combinations in the gender mismatch experiment. As for acceptability judgments, the ANOVA analysis revealed the following results. In the nominalization experiment there was a significant effect of STEM TYPE (*p* < 0.001) on acceptability ratings and interaction between STEM TYPE and CASE (*p* < 0.001); in the gender mismatch experiment, we found a significant effect of PATTERN (*p* < 0.001) on acceptability ratings; in the paucal construction experiment we observed significant effects of CONTEXT (*p* < 0.001), PATTERN (*p* < 0.001), and CASE (*p* < 0.001), and a significant CONTEXT-CASE interaction (*p* < 0.001). All statistical tests were run in the R environment.

As the hypothesis of our study concerns the connection between frequency of occurrence and acceptability judgments, for each phenomenon we shall review the results of both experiments jointly.

**FIGURE 1:**
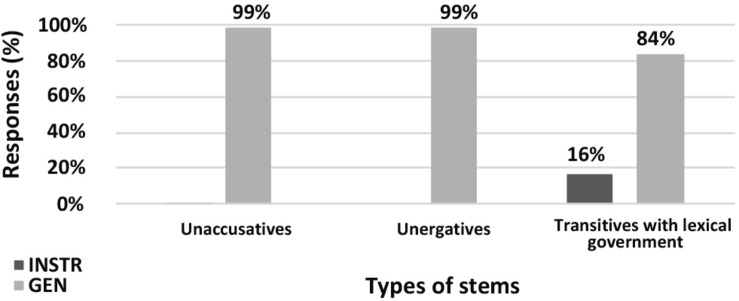
Production experiment frequencies for nominalizations. Diagram comparing the production frequencies for INSTR (dark) and GEN (light) case marking of external argument with different nominalization stems.

In the **nominalization** production experiment, both GEN and INSTR were available as case marking strategies for transitive stems with lexical government. With unergatives speakers only made use of GEN. As predicted by previous research, there was no variation in the control conditions: only INSTR was available for transitives, and with unaccusatives INSTR was rarely used (1% of answers). For transitive stems with lexical government GEN was more frequent, which aligns with the results from Pereltsvaig et al. (2018). The distribution of GEN and INSTR for different stems is presented in Figure 1.

**FIGURE 2:**
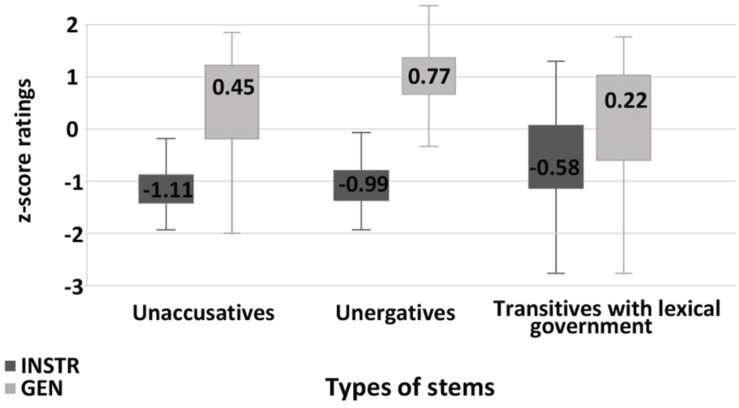
Acceptability rating for nominalizations. Box-plot comparing the ratings for INSTR (dark) and GEN (light) case marking of external argument with different nominalization stems. Error bars represent standard deviation. Acceptability ratings are *z*-score transformed.

In the nominalization judgment experiment, we observed a significant difference in acceptability rates for INSTR for different stems (Figure 2). Importantly, INSTR was significantly more acceptable with stems with lexical government than with unaccusative stems, baseline condition (Student’s *t*-test, *p* = 0.03). The acceptability scores for unergative stems did not differ significantly from the scores for unaccusative stems. That is, both production and judgment experiments contradict the suggestion that unergatives group with transitive stems with lexical government.

**FIGURE 3:**
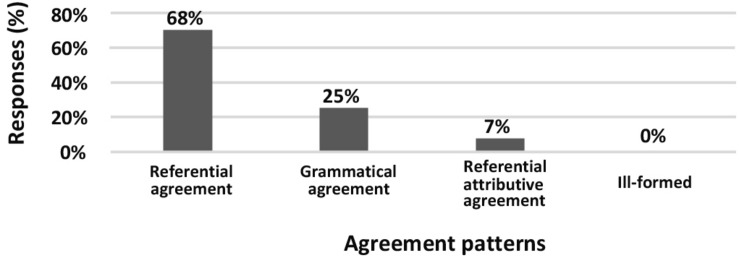
Production experiment frequencies for gender mismatch patterns. Diagram comparing the production frequencies for different agreement patterns in gender mismatch constructions.

**FIGURE 4:**
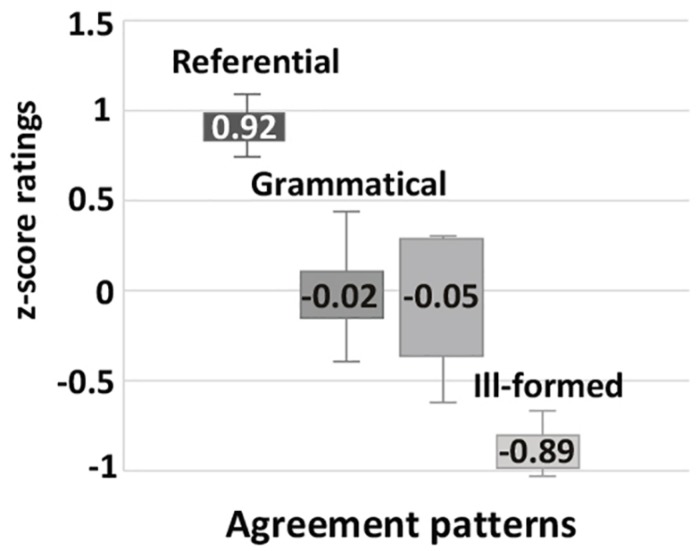
Acceptability rating for gender mismatch patterns. Box-plot comparing the ratings for different agreement patterns in gender mismatch constructions. Error bars represent standard deviation. Acceptability ratings are *z*-score transformed.

The **gender mismatch** production experiment showed that neither frequency nor judgment of patterns differ significantly for different combinations of adnominals. The most important result is that REFERENTIAL AGREEMENT was the most frequent pattern for all combinations of adjective modifiers, which supports the observations of both prescriptive grammars and formal research papers (Figure 3). The REFERENTIAL AGREEMENT pattern was also considered the most acceptable one in the acceptability judgment experiment. It was rated significantly more acceptable than GRAMMATICAL AGREEMENT and FEMININE ATTRIBUTIVE AGREEMENT (Student’s *t*-test, *p* < 0.01) (Figure 4).

The differences between the results of the two experiments appear when comparing the GRAMMATICAL AGREEMENT pattern and the FEMININE ATTRIBUTIVE AGREEMENT pattern. Although GRAMMATICAL AGREEMENT and FEMININE ATTRIBUTIVE AGREEMENT had significantly different frequencies in the production experiment (25% vs. 7%), they had statistically equal acceptability scores (raw means 2.92 vs. 2.75 and *z*-score means −0.02 vs. −0.05; Student’s *t*-test, *p* > 0.1).

**FIGURE 5:**
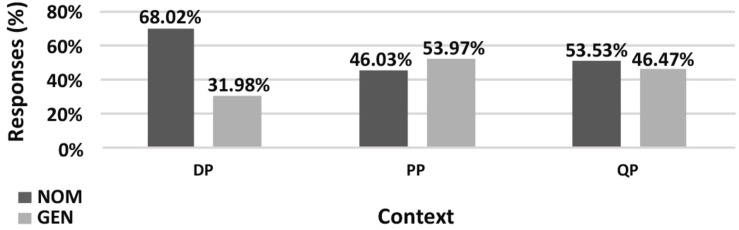
Production experiment frequencies for paucal constructions with nominalized adjectives. Diagram comparing the production frequencies for NOM (dark) and GEN (light) case marking of nominalized adjectives in paucal constructions in different contexts.

The results of the **paucal construction** experiments generally supported the hypotheses and observations reported in the previous literature. However, there are differences between the results for nominalized adjectives and those for adjectives modifying feminine nouns. In particular, for nominalized adjectives in argumental (DP) position NOM is preferred over GEN (χ^2^, *p* < 0.01), while in quantificational positions (PP and QP) both NOM and GEN are available, see Figure 5. For attributive adjectives in argumental (DP) position NOM is preferred over GEN (χ^2^, *p* < 0.01), and for attributive adjectives in quantificational positions (PP and QP) GEN is preferred over NOM (χ^2^, *p* < 0.01) (Figure 7).

**FIGURE 6:**
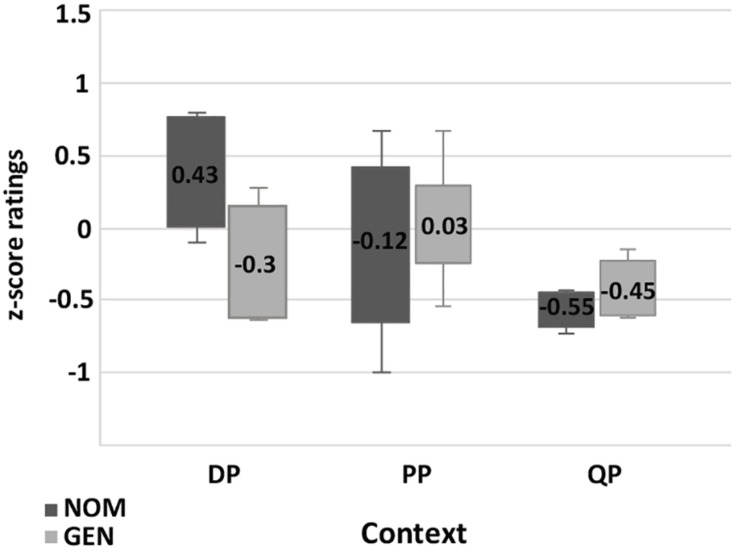
Acceptability rating for paucal constructions with nominalized adjectives. Box-plot comparing the ratings for NOM (dark) and GEN (light) case marking of nominalized adjectives in paucal constructions in different contexts. Error bars represent standard deviation. Acceptability ratings are *z*-score transformed.

**FIGURE 7:**
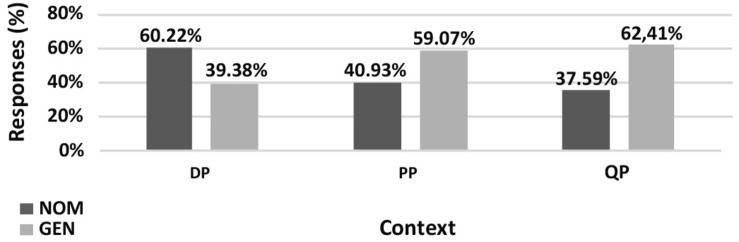
Production experiment frequencies for paucal constructions with adjectives. Diagram comparing the production frequencies for NOM (dark) and GEN (light) case marking of adjectives in paucal constructions in different contexts.

**FIGURE 8:**
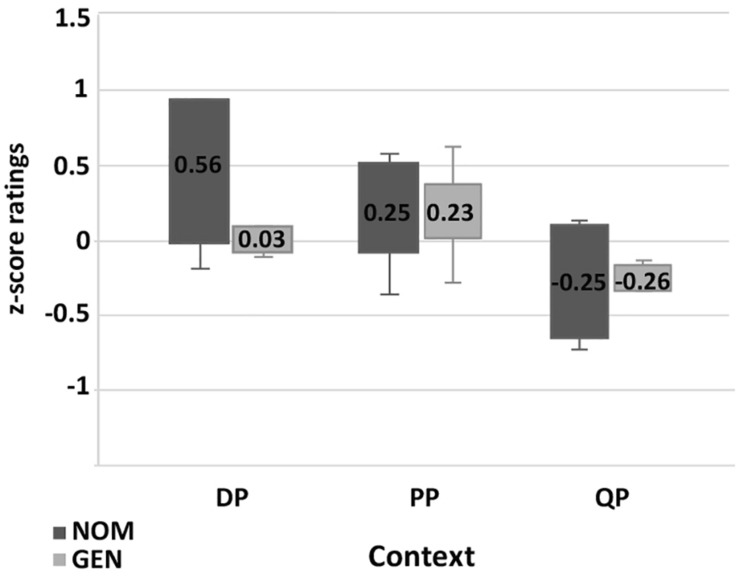
Acceptability rating for paucal constructions with adjectives. Box-plot comparing the ratings for NOM (dark) and GEN (light) case marking of adjectives in paucal constructions in different contexts. Error bars represent standard deviation. Acceptability ratings are *z*-score transformed.

In the judgment experiment for both nominalized adjectives and adjectives that modify feminine nouns in argumental (DP) position NOM is rated as significantly more acceptable than GEN (Student’s *t*-test, *p* < 0.01) (Figures 6, 8). For both types of adjectives in quantificational contexts (PP and QP) NOM and GEN have almost the same acceptability ratings (Student’s *t*-test, *p* > 0.1). This means that the judgment results support the production results in all the conditions except for attributive adjectives in quantificational contexts (PP and QP). In the latter case GEN is clearly preferred in production, but NOM and GEN have almost the same acceptability ratings.

## Discussion of the Experimental Results

As can be seen from the data analysis, the tendencies predicted theoretically are supported by the experimental data in both types of experiments. The results indicate that (i) for nominalizations derived from transitive stems with lexical government GEN is more frequent and more acceptable than INSTR, (ii) the REFERENTIAL AGREEMENT pattern is the most frequent and the most acceptable choice for gender mismatch nouns, (iii) in paucal constructions in argumental (DP) position NOM is more frequently used and is rated as more acceptable than GEN, and in paucal constructions in quantificational positions (PP and QP) NOM and GEN are both available and rated equally acceptable. However, it is worth noticing that there is no ceiling effect for any variant in the target conditions in either of the surveys.

The crucial observation is that the results of the two experiments do not necessarily coincide. In the case of gender mismatch, the two agreement patterns, GRAMMATICAL AGREEMENT and REFERENTIAL ATTRIBUTIVE AGREEMENT, are produced and rated at different levels. Similar disparities are observed in the paucal construction experiments: in quantificational context condition for adjectives there is no preference in judgments but a clear preference for GEN in usage.

The goal of our study, however, is to analyze the consistency of individual speakers over the production and perception domains. In the next section, we aim to explore whether the speakers’ evaluation of the acceptability of the alternatives is consistent with the grouping based on their actual usage in production.

### Analysis of the Consistency of Respondents

Adopting the view that grammar is probabilistic in nature presumes that frequencies of occurrence and acceptability scores are functions of the same grammatical constraints. The two domains are clearly non-identical, and the differences between the two modalities inevitably add noise and distortion to how the grammatical constraints are implemented. Hence, we assume that it is unreasonable to relate either the absolute or the relative size of differences in ratings to frequency differences. Instead, we suggest analyzing *relative directional differences*, viz. whether the *direction* of acceptability is predicted by production or vice versa. In case a respondent is consistent over pairs of experiments, we expect that in both production and judgments there will either be a preference toward one of the variants or both variants will be permitted and judged acceptable.

To measure the consistency of individual respondents, we checked whether each respondent who participated in both experiments rated the variant that she used in the production experiment as more acceptable than the alternative. In particular, we developed a metric which was computed as follows. For nominalizations and paucal constructions we registered (i) what the respondent produced, whether one or both alternatives, in a certain condition and (ii) which of the two alternatives the respondent rated as more acceptable in the very same condition. For the latter, we compared the mean values in raw format. Those cases where the mean values were equal were counted as if both variants were acceptable. The gender mismatch experiments were different from the two other sets in that they offered a choice of four major patterns. Nevertheless, there were very few cases where a respondent rated more than two patterns as equally acceptable, so there was no need to compute the metric for this phenomenon differently.

With the new metric we compared the same conditions across experiments that were conducted using different methodologies. As we are interested in comparing production and perception for the phenomena prone to variation, when making the comparison we took into account only those conditions that allowed for variation. Notice that the metric does not consider the same lexical variants, as the two types of experiments contained different numbers of stimuli.

The results of the consistency analysis are presented in Table 5. The most striking result to emerge from the data is that, on average, respondents stick to one variant in only half of the conditions that allow for variation. For instance, for the nominalizations the metric shows that in 55% of cases the answer provided to a given condition was the same in both experiments, while in 29% of cases respondents allowed both variants in one experiment but preferred only one variant in the other, and in 16% of cases the variant produced was in fact rated as the least acceptable. The figures are even more revealing for paucal constructions. Here, the production and the choice that was rated as the most acceptable coincided in only 39% of cases, while in 37% of cases both variants were allowed in one of the experiments and only one variant in the other. In 24% of cases the variant used was rated as the least acceptable.

**TABLE 5:** Relative directional difference for the three experiments.

Three strategies of choice and rating	Nominalizations	Gender mismatch	Paucal constructions
1. What is produced is rated as most acceptable	55%	57%	39%
2. One alternative in one experiment, and both in the other	29%	30%	37%
2a. Both variants in production	25%	14%	23%
2b. Both variants in judgments	4%	16%	14%
3. Different alternatives in each experiment	16%	13%	24%

*In each cell of the table we present the percentage of cases when the respondent demonstrated the given behavior toward an experimental condition. All conditions were taken from the production experiments.*

In the gender mismatch experiments, the preference for a single pattern was preserved in 57% of answers^11^. The gender mismatch experiments were different from the two other sets in that there was a choice to be made between four major patterns. Nevertheless, there were very few instances when a respondent rated more than two patterns as equally (highly) acceptable. In 30% the results partly coincided, with respondents showing more flexibility in one of the experiments than in the other. Finally, in 13% of answers respondents were inconsistent.

The consistency analysis also shows that in the nominalization and paucal construction experiments respondents were more likely to use both variants in production than in their acceptability judgments. For gender mismatch experiments these rates did not differ.

As there were several experimental items for one condition, we estimated whether respondents were more consistent within one condition in production or acceptability judgment experiments. The results of the computations are presented in Table 6. Within each experiment, we analyzed whether a respondent was consistent across different lexicalizations of a single condition.

**TABLE 6:** Consistency of respondents within one experiment with respect to one condition.

	Nominalizations		Gender mismatch		Paucal constructions	
	Production	Judgments	Production	Judgments	Production	Judgments
The same variant within one condition (is produced/rated as most acceptable)	73%	94%	85%	82%	71%	80%
Different variants within one condition (are produced/rated as most acceptable)	27%	6%	15%	18%	29%	20%

The analysis shows that in the nominalization and paucal construction experiments there was more variability within production than in acceptability judgments. In gender mismatch experiments, there was no difference in variability. Taken together the two metrics indicate paucal constructions to be more unstable than the other two phenomena: there was much more variability in the answers given in relation to paucal constructions in both production and acceptability judgment experiments.

## General Discussion

The main goal of this study was to investigate how grammatical options can be distributed in the production and perception domains of a single speaker. Specifically, we hypothesized that if grammatical knowledge is indeed probabilistic, a single speaker would be consistent across the two domains of speech, providing data that follows the same grammatical constraints in both offline production and offline perception. The stated objective determined the methodology for the study: in this paper, we reported two series of experiments which involved both production and acceptability judgments. The experimental materials were based on three types of constructions in Russian which display a certain degree of variability.

Three findings from the experiments reported above can be identified as the most important. First, the experimental data in general supports the idea of alignment between acceptability ratings and frequency of occurrence. In all three pairs of experiments, the most frequent variant coincided with the one that received the highest acceptability score (GEN for transitive nominalizations with lexical government, REFERENTIAL AGREEMENT for gender mismatch nouns, NOM for paucal constructions in argumental position). Second, the results of production experiments do not always correspond to the associated acceptability ratings, even when production and ratings are provided by the same respondents. This is the case for GRAMMATICAL AGREEMENT and REFERENTIAL ATTRIBUTIVE AGREEMENT with gender mismatch nouns and for the distribution of NOM and GEN in paucal constructions in quantificational position. Third, speakers are not consistent in choosing one variant across the two types of experiment: more variation is allowed in production experiments. Moreover, variation can be characterized from the point of view of speaker consistency: different phenomena exhibit different values for consistency measures.

### Inconsistency and the Diachronic Status of a Phenomenon

In this section, we would like to discuss the possible sources of inconsistency across the experiments. A plausible reason for inconsistency is the nature of the phenomena examined. As we are discussing variation, we are entering supposedly unstable language domains and examining constructions undergoing change. This change is to a great extent driven by the Economy Principle [also known as the Principle of Least Effort (Zipf, 1949)], viz., the tendency to economize on cognitive resources when conveying a message. In the context of historical linguistics, the Economy Principle is regarded as a trigger for grammatical change, since it is not economical to expend resources on several competing variants. As the existence of several options is not in accordance with expending less effort, it is expected either for the alternation to disappear (via the disappearance of one of the variants) or for the distribution of the variants to become fixed. Unless this state is achieved, we are observing different stages of language development. The periphery of variation, viz. those variants that are at the low end of the frequency spectrum, might indeed be (i) the residual effects of language evolution or, conversely, (ii) prerequisites for future changes. That is, inconsistency across the answers given by a single respondent in this case can be expected. What is remarkable is that the types of inconsistency observed differ, which means that the variation can be further characterized from this point of view.

In particular, for **nominalizations** INSTR case marking is reported as a rather new strategy (Pereltsvaig et al., 2018). This diachronic property serves as an explanation for the low frequency counts displayed by this variant. We suggest that due to its innovative nature the strategy is still rated as somewhat unacceptable even by those respondents who use it.

In cases of **gender mismatch**, REFERENTIAL AGREEMENT has been reported as the principal strategy since the 1970s (Muchnik, 1971; Crockett, 1976; Shvedova, 1980). However, while in production speakers predominantly follow a certain pattern, they also produce structurally possible alternatives to which they give equal scores: GRAMMATICAL AGREEMENT is still more frequent than FEMININE ATTRIBUTIVE AGREEMENT, but both variants have the same, rather low, level of acceptability. That is, the two alternative variants on the periphery are equalized when consciously considered. We hypothesize that these judgments reflect a gradual decrease in production frequency of the GRAMMATICAL AGREEMENT pattern in comparison to the favored REFERENTIAL AGREEMENT pattern^12^.

A conceptually similar situation is found for QP contexts in **paucal constructions:** in production respondents prefer one variant, but they rate both possibilities equally when perceiving them. That is, while there is a clear leader in production, judgments reveal this only partly, via the dispersion of possible answers, which is higher for the less common variant.

We suppose that the degree of coherence of the two experiments corresponds to different stages of the evolution of the variation involved. What we observe in case of gender mismatch might be the effects of the disappearance of variation. In contrast, in the case of nominalizations we see the ongoing development of a competing variant. In the case of paucal constructions, we do not have enough diachronic data to predict the direction of change; however, Economy Principle considerations suggest that variants are becoming more fixed with respect to the structural position they take up.

### Inconsistency and the Experimental Methodology

The data shows that elicited production and acceptability judgments differ with respect to how they reveal variation in language. We suggest that this inconsistency is partly dictated by the properties of the methodology used. Acceptability judgments in general show less variability. The restrictive quality of the method is revealed when analyzing whether respondents are consistent within one condition in separate experiments: within one condition the same variant is chosen more often in the judgment experiment than in the production experiment (Table 6). The question is what mechanisms behind the experimental methods involving production and perception determine the differences in the results.

As stated by Schütze (1996), an acceptability judgment is a reported perception of acceptability. It is not clear what the mechanisms are that help to bring about this percept: whether it is accumulated during the process of perceiving the sentence, while the respondent is comparing the actual percept with her expectations [e.g., as in the theory of forward action modeling by Garrod and Pickering (2013)], or whether the procedure is more complicated. Regardless of the specific percept model, we suppose that what is present in the case of judgment, and lacking in production, is reference to previous metalinguistic experience when deciding on an exact rating. We hypothesize that during the acceptability judgment experiment the respondent is referring to her previous experience, i.e., to the percepts of other sentences that she has perceived. Our idea is that this reference in itself produces a cognitive load that restricts the availability of the less activated elements. That is why this additional step leads to greater restrictiveness in comparison to production results.

Although the rating task makes the choice more restricted, we argue that the production method should not be generally preferred as more sensitive. Neither production nor judgment data provide direct access to the grammar: they add distortion of different kinds, as different sets of cognitive systems are involved in the processes of production and perception. We suggest that the two experimental methods used in this paper are sensitive to different aspects of language phenomena. In particular, elicited production is better in revealing deviations from the patterns prescribed in grammars, while acceptability judgments are better at investigating to what extent a grammatical innovation has become established in the language. The combination of production and judgment data thus allows us to estimate the directionality of ongoing changes in variability and gain access to the full distribution of variants.

This observation leads to another question, namely, how the results obtained in this paper can be extrapolated to other language phenomena that do not exhibit such variability. In this study, we examined three types of construction reported in the previous literature as involving variation. However, we doubt that one can ultimately tell where the variability ends. It might be impossible to eliminate variability and ascertain whether a phenomenon is “stable” in advance of carrying out research on it. We believe that any language phenomenon should be analyzed taking into consideration both production and judgment data, as it is potentially subject to variation of the type investigated here.

### Implications for Methodology

The way experimental methods are applied traditionally presupposes analyzing the sample as a whole and averaging out the individual differences. However, the properties of individual behavior toward a certain phenomenon might provide a glimpse of its current state.

Similar ideas are being developed in the field of research on bilingualism. The multidimensional concept of bilingualism cannot be treated as a categorical variable because bilingual experience shapes the way executive functioning is performed (Takahesu et al., 2018; De Bruin, 2019). Both production and comprehension processes adapt to the demands determined by the bilinguals’ previous language experience: for instance, Beatty-Martínez and Dussias (2017) provide supporting evidence analyzing how individuals’ production choices correlate with their code-switching strategies. The differences in how production and comprehension processes are tuned might be defined not only by language experience, but also by a set of individual-level skills such as word-decoding, working memory, and susceptibility to memory interference (Fricke et al., 2019). Rather than treating interspeaker variation as noise, an increasing number of studies propose that interspeaker variation could shed light on the linguistic architecture and how it is coordinated with other cognitive systems.

Remarkably, the results of our study suggest that linguistics could benefit from implementing an even more fine-grained approach and taking into account the behavior of each individual speaker. Differences in linguistic experience and cognitive skills supposedly should not influence the link between the production and perception domains of a single speaker. Even though respondents differ in experience with respect to certain language phenomena (e.g., poor input), we expect them to be consistent in their individual preferences across different tasks.

As the result of our study, we have devised a metric that allows us to estimate the consistency of respondents with respect to a language phenomenon in the two language domains. In particular, we have shown that inconsistency rates are far from being random, both within a single experiment and across experiments conducted with different methodologies. Importantly, the metric allows us to characterize each condition in the experiment in terms of speakers’ consistency in using a certain variant. Consequently, it can also be used within a single phenomenon for a comparative analysis of conditions. We believe that the elaborated metric can be used as a formal instrument for the description of variation and will be beneficial in studying language phenomena displaying variability. Further work is needed to investigate how far speakers can be inconsistent in the production and perception of a certain phenomenon such that the phenomenon may still be considered a part of the language system. Another interesting issue is how changes in the consistency of certain individuals’ behavior may influence the dynamics behind innovations in a language community. We leave these questions to future research.

## Conclusion

The present study investigated the correspondence between offline production and offline perception in the speech of individual speakers. In our study we focused on variation and examined three types of construction that display a certain degree of variability. As can be seen from the results, using just one experimental technique would somewhat limit our understanding of the phenomena under investigation. Our data suggest that there is a correspondence between frequency of occurrence and acceptability rates. However, this correspondence is more complicated than has been stated in previous studies: different phenomena involving variation deviate from the ideal correspondence to different extents. We have shown that the combination of two sources of data provides a fuller description for cases of intralingual variation than the use of a single method. The way the data sources conform allows us to distinguish different types of variation and define unstable language domains, and, furthermore, it can serve as an additional descriptive measure.

## Data Availability Statement

The datasets generated for this study are available on request to the corresponding author.

## Ethics Statement

The Commission for Ethics of Pushkin State Russian Language Institute confirmed that in accordance with the Legislation of the Russian Federation appropriate informed consent was obtained from each research participant or participant’s legal guardian/next of kin, the data storage was organized in accordance with the law, and the research design was in accordance with the relevant ethical standards.

## Author Contributions

AG and EL have made substantial, direct and intellectual contribution to the conception and design of the work, interpretation of data, analysis, and have both approved it for publication.

## Conflict of Interest

The authors declare that the research was conducted in the absence of any commercial or financial relationships that could be construed as a potential conflict of interest.

## Footnotes

1 In line with Schütze (1996), we use the term *grammaticality* when referring to grammar as a mental construct, and the term *acceptability* when referring to judgments.

2 The negative spectrum of acceptability was covered by sentences that were produced by first translating a set of sentences from the British National Corpus to four languages (Norwegian, Spanish, Japanese, and Chinese) and then translating them back to English using Google Translate.

3 A similar comparison of methods was reported in Klavan and Veismann (2017), which compared the performance of a corpus-based language model against the results of two rating experiments: forced-choice and Likert scale. The results of the study show that forced-choice data provides a slightly better reflection of the corpus than Likert scale data. This might result from the higher statistical power of the method. For instance, Stadthagen-González et al. (2017) compare Likert scale judgments to a 2-alternative forced-choice task combined with the Thurstone measurement model, which allows the results of comparisons to be laid out along a single interval scale. The results of the study show that with some experimental conditions a forced-choice task might yield more granular data than pure ratings.

4 Another advantage in using the examined set of phenomena is that none of them are mentioned in prescriptive grammars. This means that respondents were not influenced by prescriptive grammars and would not draw on their school knowledge of grammar when participating in the experiments.

5 We thank the reviewer for bringing this issue to our attention.

6 To the best of our knowledge there are no studies exploring how long it takes for respondents to forget linguistic stimuli. The effects of repeated exposure of linguistic stimuli have only been studied with respect to syntactic satiation, an effect whereby sentences that were initially judged ungrammatical come to be judged as acceptable. However, this phenomenon is usually studied within a single testing session (as in Francom, 2009; Hofmeister et al., 2013).

7 As proposed by Featherston (2007), asking about the *naturalness* of the stimuli avoids reference to the informant’s own production and encourages her to focus on spoken rather than written form. However, we added the opposition “good”/“bad” as a more traditional option in experimental syntax and in our opinion one which is more intuitive for naïve respondents.

8 Training sentences included both grammatical and ungrammatical sentences, which were pre-tested on a sample of 15 respondents. In order to assess their competence at completing the task, we divided the Likert scale into two halves and checked whether respondents attributed fillers to the correct half of the scale, positive or negative. In case there were mistakes in judging the training sentences, all results from the respondent in question were excluded from the analysis.

9 For descriptive purposes, the extraneous variables are indicated when counting the number of conditions. Different scholarly traditions treat extraneous variables differently: e.g., some may omit them when counting the final number of conditions. We suppose that including them makes the description of the stimuli more transparent. These variables were tested to ensure that they were not confounders.

10 Given the declared number of controlled variables, the reviewer suggested that we should address the issue of statistical power. To our knowledge, the only paper that has systematically examined the statistical power of linguistic experiments is (Sprouse and Almeida, 2017). Importantly, Sprouse and Almeida (2017) estimated the sample size requirements for obtaining 80% power for a Likert scale judgment survey. This was calculated for the lowest bound for power, as only one item per experimental condition was used. The calculations were performed using resampling simulations and information about the effect size for the phenomena taken from the previous experimental study by Sprouse et al. (2013), in which judgments were tested for a random sample of 150 phenomena from Linguistic Inquiry 2001–2010. The median effect size for that sample was a *Cohen’s d* of 1.61, which was taken as the “average effect size” in linguistic studies. Given this average effect size and the lowest bound for power, it was shown that ten participants provide 80% power for Likert scale experiments, i.e., in 80% of such cases acceptability rating differences can be detected at statistical significance. The experiments conducted in the present study significantly exceed the requirements stated in Sprouse and Almeida (2017).

11 In the gender mismatch experiment, there was more than one theoretically possible number of alternatives. Therefore, there was a methodological question concerning which situation should be recorded under the heading *usage/acceptance of both variants*: on one hand, it was important to maintain the application of a similar metric across the three sets of experiments, but on the other hand, it would be practically impossible for a respondent to use all three possible patterns within one experiment. Therefore, we recorded that *both variants* were used in a production experiment or *both variants* were rated acceptable when at least two possible variants out of three were used or rated acceptable, respectively. The properties of the gender mismatch experiment also dictate a different definition for what situations should be considered as *preference for different alternatives*. If a respondent used the REFERENTIAL AGREEMENT pattern in the production experiment but rated GRAMMATICAL AGREEMENT and REFERENTIAL ATTRIBUTIVE AGREEMENT as equally acceptable and more acceptable than REFERENTIAL AGREEMENT, this situation was counted as *preference for different alternatives* in each experiment (even though the choice was not binary).

12 Muchnik (1971) reported results from a questionnaire completed by 3,780 Russian native speakers, which showed that GRAMMATICAL AGREEMENT was chosen in 38.6% of cases, while in the current study GRAMMATICAL AGREEMENT was used 25.21% of the time. Although this is in line with our hypothesis, it may not be appropriate to compare the results of the earlier study with those produced by the current research due to differences in their design. Muchnik’s questionnaire included only two lexical variants of the combination “high adjective + noun” and two lexical variants of the combination “noun + verb,” and the questions were of the following type: *How would you say, with reference to a woman: “nice-M doctor” or “nice-F doctor”?* No context was provided for the noun phrase and the verb, and the participants’ attention was drawn toward the agreement properties, which significantly lowers the ecological validity of the survey.
