# Supplementary material for: Intralingual Variation in Acceptability Judgments and Production: Three Case Studies in Russian Grammar
Source: Front Psychol. 2020 Mar 31;11:348. doi: 10.3389/fpsyg.2020.00348 (PMC7136902; doi:10.3389/fpsyg.2020.00348)
Supplement: Supplementary file 1 [file Data_Sheet_1.PDF]

Supplementary Material

1 Introduction

Below we present the experimental stimuli. The experimental stimuli are presented for all conditions in two versions: a native Russian sentence (a) and a transliterated sentence (b).

2 Nominalization experiments

The digit stands for lexicalization. "Trans" stands for transitive stem, "trans_lex" stands for transitive stem with lexical government, "unerg" stands for unergative stem, "unacc" stands for unaccusative stem.

1.trans.a. В тот месяц армия освободила столицу, и освобождение армии/армией столицы сильно подняло боевой дух солдат.

1.trans.b. V tot mesjac armija osvobodila stolicu, i osvoboždenie armii/armiej stolicy sil'no podnjalo boevoj dux soldat.

2.trans_lex.a. В течение матча судья подыгрывал команде, а подыгрывание судьи/судьей команде строго запрещено по правилам чемпионата.

2.trans_lex.b. V tečenie matča sud'ja podygryval komande, a podygryvanie sud'i/sud'ej komande strogo zapreščeno po pravilam čempionata.

3.unerg.a. После процедуры пациент стал кашлять, и кашляние пациента/пациентом сразу насторожило лечащего врача.

3.unerg.b. Posle procedury pacient stal kašljat', i kašljanie pacienta/pacientom srazu nastorožilo lečaščego vrača.

4.unacc.a. Каждую осень бабушка приезжала к нам в город, и приезд бабушки/бабушкой всегда сопровождался вкусным и сытным застольем.

4.unacc.b. Každuju osen' babuška priezžala k nam v gorod, i priezd babuški/babuškoj vsegda soprovoždalsja vkusnym i sytnym zastol'em.

5.trans.a. Перед концертом музыкант включил микрофон, и включение музыканта/музыкантом микрофона немедленно привело к поломке динамика.

5.trans.b. Pered koncertom muzykant vključil mikrofon, i vključenie muzykanta/muzykantom mikrofona nemedlenno privelo k polomke dinamika.

6.trans_lex.a. На параде танкист управлял машиной, и управление танкиста/танкистом машиной глубоко впечатлило всех зрителей.

6.trans_lex.b. Na parade tankist upravljal mašinoj, i upravlenie tankista/tankistom mašinoj gluboko vpečatlilo vsex zritelej.

7.unerg.a. Под шум моря малыш сопел в кроватке, и сопение малыша/малышом во сне умиляло его родителей.

7.unerg.b. Pod šum morja malyš sopel v krovatke, i sopenie malyša/malyšom vo sne umiljalo ego roditelej.

8.unacc.a. Каждую ночь призрак возникал в его памяти, и возникновение призрака/призраком постоянно сводило меня с ума.

8.unacc.b. Každuju noč' prizrak voznikal v ego pamjati, i vozniknovenie prizraka/prizrakom postojanno svodilo menja s uma.

9. trans.a. Каждый вечер родители купают младенца, и купание родителей/родителями младенца непременно увеличивает расход воды.

9. trans.b. Každyj večer roditeli kupajut mladenca, i kupanie roditelej/roditeljami mladenca nepremenno uveličivaet rasxod vody.

10.trans_lex.a. В любом споре секретарь поддакивал начальнику, но поддакивание секретаря/секретарем начальнику никак не привело к кадровым перестановкам.

10.trans_lex.b. V ljubom spore sekretar' poddakival načal'niku, no poddakivanie sekretarja/sekretarem načal'niku nikak ne privelo k kadrovym perestanovkam.

11.unerg.a. Даже зимой дедушка плавает в речке, хотя плавание дедушки/дедушкой сильно беспокоит всех его родственников.

11.unerg.b. Daže zimoj deduška plavaet v rečke, xotja plavanie deduški/deduškoj sil'no bespokoit vsex ego rodstvennikov.

12.unacc.a. В этом году президент прибыл на матч, и прибытие президента/президентом сразу привлекло много внимания к чемпионату.

12.unacc.b. V jetom godu prezident pribyl na matč, i pribytie prezidenta/prezidentom srazu privleklo mnogo vnimanija k čempionatu.

13.trans.a. На фестивале профессор читал лекцию, и чтение профессора/профессором лекции сильно впечатлило всех слушателей.

13.trans.b. Na festivale professor čital lekciju, i čtenie professora/professorom lekcii sil'no vpečatlilo vsex slušatelej.

14.trans_lex.a. Своим поступком пешеход отомстил водителю, но мщение пешехода/пешеходом водителю в итоге привело к усугублению конфликта.

14.trans_lex.b. Svoim postupkom pešexod otomstil voditelju, no mščenie pešexoda/pešexodom voditelju v itoge privelo k usugubleniju konflikta.

15.unerg.a. Из-за астмы один зритель раскашлялся, и кашляние зрителя/зрителем очень смутило его соседей по ложе.

15.unerg.b. Iz-za astmy odin zritel' raskašljalsja, i kašljanie zritelja/zritelem očen' smutilo ego sosedej po lože.

16.unacc.a. На этой неделе учитель заболел, а заболевание учителя/учителей иногда ведет к закрытию школы на карантин.

16.unacc.b. Na jetoj nedele učitel' zabolel, a zabolevanie učitelja/učitelej inogda vedet k zakrytiju školy na karantin.

3 Gender mismatch experiments

The first digit stands for combination of adnominals; the second digit stands for lexicalization.

1.1.a. Всю ночь Тане не удалось сомкнуть глаз: наш ответственный проектный менеджер готовил презентацию рекламной кампании для радиохолдинга.

1.1.b. Vsju noč' Tane ne udalos' somknut' glaz: naš otvetstvennyj proektnyj menedžer gotovil prezentaciju reklamnoj kampanii dlja radioxoldinga.

1.2.a. Сегодня надо поздравить Анну Михайловну: мой преуспевающий страховой агент получил премию за успешную продажу полисов.

1.2.b. Segodnja nado pozdravit' Annu Mixajlovnu: moj preuspevajuščij straxovoj agent polučil premiju za uspešnuju prodažu polisov.

1.3.a. Вчера Ивановой вручили премию: наш самобытный театральный режиссёр представил спектакль на престижном фестивале.

1.3.b. Včera Ivanovoj vručili premiju: naš samobytnyj teatral'nyj režissjor predstavil spektakl' na prestižnom festivale.

1.4.a. Победой на выборах мы обязаны Ирине: наш начинающий политический технолог продвигал кандидата весьма успешно.

1.4.b. Pobedoj na vyborax my objazany Irine: naš načinajuščij političeskij texnolog prodvigal kandidata ves'ma uspešno.

1.5.a. У Елизаветы Геннадьевны однозначно кулинарный талант: ваш одаренный старший повар подготовил меню для важного банкета за сутки.

1.5.b. U Elizavety Gennad'evny odnoznačno kulinarnyj talant: vaš odarennyj staršij povar podgotovil menju dlja važnogo banketa za sutki.

1.6.a. Процветанием компании мы обязаны Анне Викторовне: наш талантливый исполнительный директор обеспечивал участие в крупнейших тендерах города.

1.6.b. Procvetaniem kompanii my objazany Anne Viktorovne: naš talantlivyj ispolnitel'nyj direktor obespečival učastie v krupnejšix tenderax goroda.

1.7.a. Я обратилась за помощью к Виктории Павловне: мой опытный художественный руководитель подобрал артистов на проект в течение недели.

1.7.b. Ja obratilas' za pomošč'ju k Viktorii Pavlovne: moj opytnyj xudožestvennyj rukovoditel' podobral artistov na proekt v tečenie nedeli.

1.8.a. Слухи о работоспособности Марины преувеличены: твой известный ландшафтный дизайнер создавал концепцию парка целый месяц.

1.8.b. Sluxi o rabotosposobnosti Mariny preuveličeny: tvoj izvestnyj landšaftnyj dizajner sozdaval koncepciju parka celyj mesjac.

1.9.a. Сегодня у Марины состоялся дебют на телевидении: наш ведущий финансовый аналитик представил прогноз цен на нефть в передаче «Экономика».

1.9.b. Segodnja u Mariny sostojalsja debjut na televidenii: naš veduščij finansovyj analitik predstavil prognoz cen na neft' v peredače «Jekonomika».

1.10.a. Как у Ольги Александровны получается все успевать: твой выдающийся главный редактор написал роман о работе в издательстве.

1.10.b. Kak u Ol'gi Aleksandrovny polučaetsja vse uspevat': tvoj vydajuščijsja glavnyj redaktor napisal roman o rabote v izdatel'stve.

1.11.a. Жители выразили свою признательность Евгении Владимировне: наш трудолюбивый главный архитектор проводил реставрацию центральных районов Волгограда.

1.11.b. Žiteli vyrazili svoju priznatel'nost' Evgenii Vladimirovne: naš trudoljubivyj glavnyj arxitektor provodil restavraciju central'nyx rajonov Volgograda.

1.12.a. В мире художественной гимнастики все знают Ирину Александровну: ваш знаменитый старший тренер вырастил поколение талантливых спортсменок.

1.12.b. V mire xudožestvennoj gimnastiki vse znajut Irinu Aleksandrovnu: vaš znamenityj staršij trener vyrastil pokolenie talantlivyx sportsmenok.

1.13.a. У меня не получилось вчера попасть на прием к Ивановой: мой знакомый зубной врач принимал пациентов только по предварительной записи.

1.13.b. U menja ne polučilos' včera popast' na priem k Ivanovoj: moj znakomyj zubnoj vrač prinimal pacientov tol'ko po predvaritel'noj zapisi.

1.14.a. Инне Аркадьевне известны все особенности этой стройки: наш успешный горный инженер разрабатывал вентиляцию для этой шахты год назад.

1.14.b. Inne Arkad'evne izvestny vse osobennosti jetoj strojki: naš uspešnyj gornyj inžener razrabatyval ventiljaciju dlja jetoj šaxty god nazad.

1.15.a. У Евгении Львовны большой опыт в общении с иностранцами: ваш одаренный учёный секретарь организовывал конференцию по индоиранским языкам в прошлом году.

1.15.b. U Evgenii L'vovny bol'šoj opyt v obščenii s inostrancami: vaš odarennyj učjonyj sekretar' organizovyval konferenciju po indoiranskim jazykam v prošlom godu.

1.16.a. В нашем отделе много учеников Ольги Петровны: наш грамотный системный программист руководил стажерами компании многие годы.

1.16.b. V našem otdele mnogo učenikov Ol'gi Petrovny: naš gramotnyj sistemnyj programmist rukovodil stažerami kompanii mnogie gody.

2.1.a. Всю ночь Тане не удалось сомкнуть глаз: наш ответственный менеджер готовил презентацию рекламной кампании для радиохолдинга.

2.1.b. Vsju noč' Tane ne udalos' somknut' glaz: naš otvetstvennyj menedžer gotovil prezentaciju reklamnoj kampanii dlja radioxoldinga.

2.2.a. Сегодня надо поздравить Анну Михайловну: мой преуспевающий агент получил премию за успешную продажу полисов.

2.2.b. Segodnja nado pozdravit' Annu Mixajlovnu: moj preuspevajuščij agent polučil premiju za uspešnuju prodažu polisov.

2.3.a. Вчера Ивановой вручили премию: наш самобытный режиссёр представил спектакль на престижном фестивале.

2.3.b. Včera Ivanovoj vručili premiju: naš samobytnyj režissjor predstavil spektakl' na prestižnom festival.

2.4.a. Победой на выборах мы обязаны Ирине: наш начинающий технолог продвигал кандидата весьма успешно.

2.4.b. Pobedoj na vyborax my objazany Irine: naš načinajuščij texnolog prodvigal kandidata ves'ma uspešno.

2.5.a. У Елизаветы Геннадьевны однозначно кулинарный талант: ваш одаренный повар подготовил меню для важного банкета за сутки.

2.5.b. U Elizavety Gennad'evny odnoznačno kulinarnyj talant: vaš odarennyj povar podgotovil menju dlja važnogo banketa za sutki.

2.6.a. Процветанием компании мы обязаны Анне Викторовне: наш талантливый директор обеспечивал участие в крупнейших тендерах города.

2.6.b. Procvetaniem kompanii my objazany Anne Viktorovne: naš talantlivyj direktor obespečival učastie v krupnejšix tenderax goroda.

2.7.a. Я обратилась за помощью к Виктории Павловне: мой опытный руководитель подобрал артистов на проект в течение недели.

2.7.b. Ja obratilas' za pomošč'ju k Viktorii Pavlovne: moj opytnyj rukovoditel' podobral artistov na proekt v tečenie nedeli.

2.8.a. Слухи о работоспособности Марины преувеличены: твой известный дизайнер создавал концепцию парка целый месяц.

2.8.b. Sluxi o rabotosposobnosti Mariny preuveličeny: tvoj izvestnyj dizajner sozdaval koncepciju parka celyj mesjac.

2.9.a. Сегодня у Марины состоялся дебют на телевидении: наш ведущий аналитик представил прогноз цен на нефть в передаче «Экономика».

2.9.b. Segodnja u Mariny sostojalsja debjut na televidenii: naš veduščij analitik predstavil prognoz cen na neft' v peredače «Jekonomika».

2.10.a. Как у Ольги Александровны получается все успевать: твой выдающийся редактор написал роман о работе в издательстве.

2.10.b. Kak u Ol'gi Aleksandrovny polučaetsja vse uspevat': tvoj vydajuščijsja redaktor napisal roman o rabote v izdatel'stve.

2.11.a. Жители выразили свою признательность Евгении Владимировне: наш трудолюбивый архитектор проводил реставрацию центральных районов Волгограда.

2.11.b. Žiteli vyrazili svoju priznatel'nost' Evgenii Vladimirovne: naš trudoljubivyj arxitektor provodil restavraciju central'nyx rajonov Volgograda.

2.12.a. В мире художественной гимнастики все знают Ирину Александровну: ваш знаменитый тренер вырастил поколение талантливых спортсменок.

2.12.b. V mire xudožestvennoj gimnastiki vse znajut Irinu Aleksandrovnu: vaš znamenityj trener vyrastil pokolenie talantlivyx sportsmenok.

2.13.a. У меня не получилось вчера попасть на прием к Ивановой: мой знакомый врач принимал пациентов только по предварительной записи.

2.13.b. U menja ne polučilos' včera popast' na priem k Ivanovoj: moj znakomyj vrač prinimal pacientov tol'ko po predvaritel'noj zapisi.

2.14.a. Инне Аркадьевне известны все особенности этой стройки: наш успешный инженер разрабатывал вентиляцию для этой шахты год назад.

2.14.b. Inne Arkad'evne izvestny vse osobennosti jetoj strojki: naš uspešnyj inžener razrabatyval ventiljaciju dlja jetoj šaxty god nazad.

2.15.a. У Евгении Львовны большой опыт в общении с иностранцами: ваш одаренный секретарь организовывал конференцию по индоиранским языкам в прошлом году.

2.15.b. U Evgenii L'vovny bol'šoj opyt v obščenii s inostrancami: vaš odarennyj sekretar' organizovyval konferenciju po indoiranskim jazykam v prošlom godu.

2.16.a. В нашем отделе много учеников Ольги Петровны: наш грамотный программист руководил стажерами компании многие годы.

2.16.b. V našem otdele mnogo učenikov Ol'gi Petrovny: naš gramotnyj programmist rukovodil stažerami kompanii mnogie gody.

3.1.a. Всю ночь Тане не удалось сомкнуть глаз: наш проектный менеджер готовил презентацию рекламной кампании для радиохолдинга.

3.1.b. Vsju noč' Tane ne udalos' somknut' glaz: naš proektnyj menedžer gotovil prezentaciju reklamnoj kampanii dlja radioxoldinga.

3.2.a. Сегодня надо поздравить Анну Михайловну: мой страховой агент получил премию за успешную продажу полисов.

3.2.b. Segodnja nado pozdravit' Annu Mixajlovnu: moj straxovoj agent polučil premiju za uspešnuju prodažu polisov.

3.3.a. Вчера Ивановой вручили премию: наш театральный режиссёр представил спектакль на престижном фестивале.

3.3.b. Včera Ivanovoj vručili premiju: naš teatral'nyj režissjor predstavil spektakl' na prestižnom festival.

3.4.a. Победой на выборах мы обязаны Ирине: наш политический технолог продвигал кандидата весьма успешно.

3.4.b. Pobedoj na vyborax my objazany Irine: naš političeskij texnolog prodvigal kandidata ves'ma uspešno.

3.5.a. У Елизаветы Геннадьевны однозначно кулинарный талант: ваш старший повар подготовил меню для важного банкета за сутки.

3.5.b. U Elizavety Gennad'evny odnoznačno kulinarnyj talant: vaš staršij povar podgotovil menju dlja važnogo banketa za sutki.

3.6.a. Процветанием компании мы обязаны Анне Викторовне: наш исполнительный директор обеспечивал участие в крупнейших тендерах города.

3.6.b. Procvetaniem kompanii my objazany Anne Viktorovne: naš ispolnitel'nyj direktor obespečival učastie v krupnejšix tenderax goroda.

3.7.a. Я обратилась за помощью к Виктории Павловне: мой художественный руководитель подобрал артистов на проект в течение недели.

3.7.b. Ja obratilas' za pomošč'ju k Viktorii Pavlovne: moj xudožestvennyj rukovoditel' podobral artistov na proekt v tečenie nedeli.

3.8.a. Слухи о работоспособности Марины преувеличены: твой ландшафтный дизайнер создавал концепцию парка целый месяц.

3.8.b. Sluxi o rabotosposobnosti Mariny preuveličeny: tvoj landšaftnyj dizajner sozdaval koncepciju parka celyj mesjac.

3.9.a. Сегодня у Марины состоялся дебют на телевидении: наш финансовый аналитик представил прогноз цен на нефть в передаче «Экономика».

3.9.b. Segodnja u Mariny sostojalsja debjut na televidenii: naš finansovyj analitik predstavil prognoz cen na neft' v peredače «Jekonomika».

3.10.a. Как у Ольги Александровны получается все успевать: твой главный редактор написал роман о работе в издательстве.

3.10.b. Kak u Ol'gi Aleksandrovny polučaetsja vse uspevat': tvoj glavnyj redaktor napisal roman o rabote v izdatel'stve.

3.11.a. Жители выразили свою признательность Евгении Владимировне: наш главный архитектор проводил реставрацию центральных районов Волгограда.

3.11.b. Žiteli vyrazili svoju priznatel'nost' Evgenii Vladimirovne: naš glavnyj arxitektor provodil restavraciju central'nyx rajonov Volgograda.

3.12.a. В мире художественной гимнастики все знают Ирину Александровну: ваш старший тренер вырастил поколение талантливых спортсменок.

3.12.b. V mire xudožestvennoj gimnastiki vse znajut Irinu Aleksandrovnu: vaš staršij trener vyrastil pokolenie talantlivyx sportsmenok.

3.13.a. У меня не получилось вчера попасть на прием к Ивановой: мой зубной врач принимал пациентов только по предварительной записи.

3.13.b. U menja ne polučilos' včera popast' na priem k Ivanovoj: moj zubnoj vrač prinimal pacientov tol'ko po predvaritel'noj zapisi.

3.14.a. Инне Аркадьевне известны все особенности этой стройки: наш горный инженер разрабатывал вентиляцию для этой шахты год назад.

3.14.b. Inne Arkad'evne izvestny vse osobennosti jetoj strojki: naš gornyj inžener razrabatyval ventiljaciju dlja jetoj šaxty god nazad.

3.15.a. У Евгении Львовны большой опыт в общении с иностранцами: ваш учёный секретарь организовывал конференцию по индоиранским языкам в прошлом году.

3.15.b. U Evgenii L'vovny bol'šoj opyt v obščenii s inostrancami: vaš učjonyj sekretar' organizovyval konferenciju po indoiranskim jazykam v prošlom godu.

3.16.a. В нашем отделе много учеников Ольги Петровны: наш системный программист руководил стажерами компании многие годы.

3.16.b. V našem otdele mnogo učenikov Ol'gi Petrovny: naš sistemnyj programmist rukovodil stažerami kompanii mnogie gody.

4.1.a. Всю ночь Тане не удалось сомкнуть глаз: наш менеджер готовил презентацию рекламной кампании для радиохолдинга.

4.1.b. Vsju noč' Tane ne udalos' somknut' glaz: naš menedžer gotovil prezentaciju reklamnoj kampanii dlja radioxoldinga.

4.2.a. Сегодня надо поздравить Анну Михайловн:у мой агент получил премию за успешную продажу полисов.

4.2.b. Segodnja nado pozdravit' Annu Mixajlovn:u moj agent polučil premiju za uspešnuju prodažu polisov.

4.3.a. Вчера Ивановой вручили премию: наш режиссёр представил спектакль на престижном фестивале.

4.3.b. Včera Ivanovoj vručili premiju: naš režissjor predstavil spektakl' na prestižnom festival.

4.4.a. Победой на выборах мы обязаны Ирине: наш технолог продвигал кандидата весьма успешно.

4.4.b. Pobedoj na vyborax my objazany Irine: naš texnolog prodvigal kandidata ves'ma uspešno.

4.5.a. У Елизаветы Геннадьевны однозначно кулинарный талант: ваш старший повар подготовил меню для важного банкета за сутки.

4.5.b. U Elizavety Gennad'evny odnoznačno kulinarnyj talant: vaš staršij povar podgotovil menju dlja važnogo banketa za sutki.

4.6.a. Процветанием компании мы обязаны Анне Викторовне: наш директор обеспечивал участие в крупнейших тендерах города.

4.6.b. Procvetaniem kompanii my objazany Anne Viktorovne: naš direktor obespečival učastie v krupnejšix tenderax goroda.

4.7.a. Я обратилась за помощью к Виктории Павловне: мой руководитель подобрал артистов на проект в течение недели.

4.7.b. Ja obratilas' za pomošč'ju k Viktorii Pavlovne: moj rukovoditel' podobral artistov na proekt v tečenie nedeli.

4.8.a. Слухи о работоспособности Марины преувеличены: твой дизайнер создавал концепцию парка целый месяц.

4.8.b. Sluxi o rabotosposobnosti Mariny preuveličeny: tvoj dizajner sozdaval koncepciju parka celyj mesjac.

4.9.a. Сегодня у Марины состоялся дебют на телевидении: наш аналитик представил прогноз цен на нефть в передаче «Экономика».

4.9.b. Segodnja u Mariny sostojalsja debjut na televidenii: naš analitik predstavil prognoz cen na neft' v peredače «Jekonomika».

4.10.a. Как у Ольги Александровны получается все успевать: твой редактор написал роман о работе в издательстве.

4.10.b. Kak u Ol'gi Aleksandrovny polučaetsja vse uspevat': tvoj redaktor napisal roman o rabote v izdatel'stve.

4.11.a. Жители выразили свою признательность Евгении Владимировне: наш архитектор проводил реставрацию центральных районов Волгограда.

4.11.b. Žiteli vyrazili svoju priznatel'nost' Evgenii Vladimirovne: naš arxitektor provodil restavraciju central'nyx rajonov Volgograda.

4.12.a. В мире художественной гимнастики все знают Ирину Александровну: ваш тренер вырастил поколение талантливых спортсменок.

4.12.b. V mire xudožestvennoj gimnastiki vse znajut Irinu Aleksandrovnu: vaš trener vyrastil pokolenie talantlivyx sportsmenok.

4.13.a. У меня не получилось вчера попасть на прием к Ивановой: мой врач принимал пациентов только по предварительной записи.

4.13.b. U menja ne polučilos' včera popast' na priem k Ivanovoj: moj vrač prinimal pacientov tol'ko po predvaritel'noj zapisi.

4.14.a. Инне Аркадьевне известны все особенности этой стройки: наш инженер разрабатывал вентиляцию для этой шахты год назад.

4.14.b. Inne Arkad'evne izvestny vse osobennosti jetoj strojki: naš inžener razrabatyval ventiljaciju dlja jetoj šaxty god nazad.

4.15.a. У Евгении Львовны большой опыт в общении с иностранцами: ваш секретарь организовывал конференцию по индоиранским языкам в прошлом году.

4.15.b. U Evgenii L'vovny bol'šoj opyt v obščenii s inostrancami: vaš sekretar' organizovyval konferenciju po indoiranskim jazykam v prošlom godu.

4.16.a. В нашем отделе много учеников Ольги Петровны: наш программист руководил стажерами компании многие годы.

4.16.b. V našem otdele mnogo učenikov Ol'gi Petrovny: naš programmist rukovodil stažerami kompanii mnogie gody.

5.1.a. Всю ночь Тане не удалось сомкнуть глаз: ответственный проектный менеджер готовил презентацию рекламной кампании для радиохолдинга.

5.1.b. Vsju noč' Tane ne udalos' somknut' glaz: otvetstvennyj proektnyj menedžer gotovil prezentaciju reklamnoj kampanii dlja radioxoldinga.

5.2.a. Сегодня надо поздравить Анну Михайловну: преуспевающий страховой агент получил премию за успешную продажу полисов.

5.2.b. Segodnja nado pozdravit' Annu Mixajlovnu: preuspevajuščij straxovoj agent polučil premiju za uspešnuju prodažu polisov.

5.3.a. Вчера Ивановой вручили премию самобытный: театральный режиссёр представил спектакль на престижном фестивале.

5.3.b. Včera Ivanovoj vručili premiju samobytnyj: teatral'nyj režissjor predstavil spektakl' na prestižnom festival.

5.4.a. Победой на выборах мы обязаны Ирине: начинающий политический технолог продвигал кандидата весьма успешно.

5.4.b. Pobedoj na vyborax my objazany Irine: načinajuščij političeskij texnolog prodvigal kandidata ves'ma uspešno.

5.5.a. У Елизаветы Геннадьевны однозначно кулинарный талант: одаренный старший повар подготовил меню для важного банкета за сутки.

5.5.b. U Elizavety Gennad'evny odnoznačno kulinarnyj talant: odarennyj staršij povar podgotovil menju dlja važnogo banketa za sutki.

5.6.a. Процветанием компании мы обязаны Анне Викторовне: талантливый исполнительный директор обеспечивал участие в крупнейших тендерах города.

5.6.b. Procvetaniem kompanii my objazany Anne Viktorovne: talantlivyj ispolnitel'nyj direktor obespečival učastie v krupnejšix tenderax goroda.

5.7.a. Я обратилась за помощью к Виктории Павловне: опытный художественный руководитель подобрал артистов на проект в течение недели.

5.7.b. Ja obratilas' za pomošč'ju k Viktorii Pavlovne: opytnyj xudožestvennyj rukovoditel' podobral artistov na proekt v tečenie nedeli.

5.8.a. Слухи о работоспособности Марины преувеличены: известный ландшафтный дизайнер создавал концепцию парка целый месяц.

5.8.b. Sluxi o rabotosposobnosti Mariny preuveličeny: izvestnyj landšaftnyj dizajner sozdaval koncepciju parka celyj mesjac.

5.9.a. Сегодня у Марины состоялся дебют на телевидении: ведущий финансовый аналитик представил прогноз цен на нефть в передаче «Экономика».

5.9.b. Segodnja u Mariny sostojalsja debjut na televidenii: veduščij finansovyj analitik predstavil prognoz cen na neft' v peredače «Jekonomika».

5.10.a. Как у Ольги Александровны получается все успевать: выдающийся главный редактор написал роман о работе в издательстве.

5.10.b. Kak u Ol'gi Aleksandrovny polučaetsja vse uspevat': vydajuščijsja glavnyj redaktor napisal roman o rabote v izdatel'stve.

5.11.a. Жители выразили свою признательность Евгении Владимировне: трудолюбивый главный архитектор проводил реставрацию центральных районов Волгограда.

5.11.b. Žiteli vyrazili svoju priznatel'nost' Evgenii Vladimirovne: trudoljubivyj glavnyj arxitektor provodil restavraciju central'nyx rajonov Volgograda.

5.12.a. В мире художественной гимнастики все знают Ирину Александровну: знаменитый старший тренер вырастил поколение талантливых спортсменок.

5.12.b. V mire xudožestvennoj gimnastiki vse znajut Irinu Aleksandrovnu: znamenityj staršij trener vyrastil pokolenie talantlivyx sportsmenok.

5.13.a. У меня не получилось вчера попасть на прием к Ивановой: знакомый зубной врач принимал пациентов только по предварительной записи.

5.13.b. U menja ne polučilos' včera popast' na priem k Ivanovoj: znakomyj zubnoj vrač prinimal pacientov tol'ko po predvaritel'noj zapisi.

5.14.a. Инне Аркадьевне известны все особенности этой стройки: успешный горный инженер разрабатывал вентиляцию для этой шахты год назад.

5.14.b. Inne Arkad'evne izvestny vse osobennosti jetoj strojki: uspešnyj gornyj inžener razrabatyval ventiljaciju dlja jetoj šaxty god nazad.

5.15.a. У Евгении Львовны большой опыт в общении с иностранцами: одаренный учёный секретарь организовывал конференцию по индоиранским языкам в прошлом году.

5.15.b. U Evgenii L'vovny bol'šoj opyt v obščenii s inostrancami: odarennyj učjonyj sekretar' organizovyval konferenciju po indoiranskim jazykam v prošlom godu.

5.16.a. В нашем отделе много учеников Ольги Петровны: грамотный системный программист руководил стажерами компании многие годы.

5.16.b. V našem otdele mnogo učenikov Ol'gi Petrovny: gramotnyj sistemnyj programmist rukovodil stažerami kompanii mnogie gody.

6.1.a. Всю ночь Тане не удалось сомкнуть глаз: ответственный менеджер готовил презентацию рекламной кампании для радиохолдинга.

6.1.b. Vsju noč' Tane ne udalos' somknut' glaz: otvetstvennyj menedžer gotovil prezentaciju reklamnoj kampanii dlja radioxoldinga.

6.2.a. Сегодня надо поздравить Анну Михайловну: преуспевающий агент получил премию за успешную продажу полисов.

6.2.b. Segodnja nado pozdravit' Annu Mixajlovnu: preuspevajuščij agent polučil premiju za uspešnuju prodažu polisov.

6.3.a. Вчера Ивановой вручили премию: самобытный режиссёр представил спектакль на престижном фестивале.

6.3.b. Včera Ivanovoj vručili premiju: samobytnyj režissjor predstavil spektakl' na prestižnom festival.

6.4.a. Победой на выборах мы обязаны Ирине: начинающий технолог продвигал кандидата весьма успешно.

6.4.b. Pobedoj na vyborax my objazany Irine: načinajuščij texnolog prodvigal kandidata ves'ma uspešno.

6.5.a. У Елизаветы Геннадьевны однозначно кулинарный талант: одаренный повар подготовил меню для важного банкета за сутки.

6.5.b. U Elizavety Gennad'evny odnoznačno kulinarnyj talant: odarennyj povar podgotovil menju dlja važnogo banketa za sutki.

6.6.a. Процветанием компании мы обязаны Анне Викторовне: талантливый директор обеспечивал участие в крупнейших тендерах города.

6.6.b. Procvetaniem kompanii my objazany Anne Viktorovne: talantlivyj direktor obespečival učastie v krupnejšix tenderax goroda.

6.7.a. Я обратилась за помощью к Виктории Павловне: опытный руководитель подобрал артистов на проект в течение недели.

6.7.b. Ja obratilas' za pomošč'ju k Viktorii Pavlovne: opytnyj rukovoditel' podobral artistov na proekt v tečenie nedeli.

6.8.a. Слухи о работоспособности Марины преувеличены: известный дизайнер создавал концепцию парка целый месяц.

6.8.b. Sluxi o rabotosposobnosti Mariny preuveličeny: izvestnyj dizajner sozdaval koncepciju parka celyj mesjac.

6.9.a. Сегодня у Марины состоялся дебют на телевидении: ведущий аналитик представил прогноз цен на нефть в передаче «Экономика».

6.9.b. Segodnja u Mariny sostojalsja debjut na televidenii: veduščij analitik predstavil prognoz cen na neft' v peredače «Jekonomika».

6.10.a. Как у Ольги Александровны получается все успевать: выдающийся редактор написал роман о работе в издательстве.

6.10.b. Kak u Ol'gi Aleksandrovny polučaetsja vse uspevat': vydajuščijsja redaktor napisal roman o rabote v izdatel'stve.

6.11.a. Жители выразили свою признательность Евгении Владимировне: трудолюбивый архитектор проводил реставрацию центральных районов Волгограда.

6.11.b. Žiteli vyrazili svoju priznatel'nost' Evgenii Vladimirovne: trudoljubivyj arxitektor provodil restavraciju central'nyx rajonov Volgograda.

6.12.a. В мире художественной гимнастики все знают Ирину Александровну: знаменитый тренер вырастил поколение талантливых спортсменок.

6.12.b. V mire xudožestvennoj gimnastiki vse znajut Irinu Aleksandrovnu: znamenityj trener vyrastil pokolenie talantlivyx sportsmenok.

6.13.a. У меня не получилось вчера попасть на прием к Ивановой: знакомый врач принимал пациентов только по предварительной записи.

6.13.b. U menja ne polučilos' včera popast' na priem k Ivanovoj: znakomyj vrač prinimal pacientov tol'ko po predvaritel'noj zapisi.

6.14.a. Инне Аркадьевне известны все особенности этой стройки: успешный инженер разрабатывал вентиляцию для этой шахты год назад.

6.14.b. Inne Arkad'evne izvestny vse osobennosti jetoj strojki: uspešnyj inžener razrabatyval ventiljaciju dlja jetoj šaxty god nazad.

6.15.a. У Евгении Львовны большой опыт в общении с иностранцами: одаренный секретарь организовывал конференцию по индоиранским языкам в прошлом году.

6.15.b. U Evgenii L'vovny bol'šoj opyt v obščenii s inostrancami: odarennyj sekretar' organizovyval konferenciju po indoiranskim jazykam v prošlom godu.

6.16.a. В нашем отделе много учеников Ольги Петровны: грамотный программист руководил стажерами компании многие годы.

6.16.b. V našem otdele mnogo učenikov Ol'gi Petrovny: gramotnyj programmist rukovodil stažerami kompanii mnogie gody.

7.1.a. Всю ночь Тане не удалось сомкнуть глаз: проектный менеджер готовил презентацию рекламной кампании для радиохолдинга.

7.1.b. Vsju noč' Tane ne udalos' somknut' glaz: proektnyj menedžer gotovil prezentaciju reklamnoj kampanii dlja radioxoldinga.

7.2.a. Сегодня надо поздравить Анну Михайловну: страховой агент получил премию за успешную продажу полисов.

7.2.b. Segodnja nado pozdravit' Annu Mixajlovnu: straxovoj agent polučil premiju za uspešnuju prodažu polisov.

7.3.a. Вчера Ивановой вручили премию: театральный режиссёр представил спектакль на престижном фестивале.

7.3.b. Včera Ivanovoj vručili premiju: teatral'nyj režissjor predstavil spektakl' na prestižnom festivale.

7.4.a. Победой на выборах мы обязаны Ирине: политический технолог продвигал кандидата весьма успешно.

7.4.b. Pobedoj na vyborax my objazany Irine: političeskij texnolog prodvigal kandidata ves'ma uspešno

7.5.a. У Елизаветы Геннадьевны однозначно кулинарный талант: старший повар подготовил меню для важного банкета за сутки.

7.5.b. U Elizavety Gennad'evny odnoznačno kulinarnyj talant: staršij povar podgotovil menju dlja važnogo banketa za sutki.

7.6.a. Процветанием компании мы обязаны Анне Викторовне: исполнительный директор обеспечивал участие в крупнейших тендерах города.

7.6.b. Procvetaniem kompanii my objazany Anne Viktorovne: ispolnitel'nyj direktor obespečival učastie v krupnejšix tenderax goroda.

7.7.a. Я обратилась за помощью к Виктории Павловне: художественный руководитель подобрал артистов на проект в течение недели.

7.7.b. Ja obratilas' za pomošč'ju k Viktorii Pavlovne: xudožestvennyj rukovoditel' podobral artistov na proekt v tečenie nedeli.

7.8.a. Слухи о работоспособности Марины преувеличены: ландшафтный дизайнер создавал концепцию парка целый месяц.

7.8.b. Sluxi o rabotosposobnosti Mariny preuveličeny: landšaftnyj dizajner sozdaval koncepciju parka celyj mesjac.

7.9.a. Сегодня у Марины состоялся дебют на телевидении: финансовый аналитик представил прогноз цен на нефть в передаче «Экономика».

7.9.b. Segodnja u Mariny sostojalsja debjut na televidenii: finansovyj analitik predstavil prognoz cen na neft' v peredače «Jekonomika».

7.10.a. Как у Ольги Александровны получается все успевать: главный редактор написал роман о работе в издательстве.

7.10.b. Kak u Ol'gi Aleksandrovny polučaetsja vse uspevat': glavnyj redaktor napisal roman o rabote v izdatel'stve.

7.11.a. Жители выразили свою признательность Евгении Владимировне: главный архитектор проводил реставрацию центральных районов Волгограда.

7.11.b. Žiteli vyrazili svoju priznatel'nost' Evgenii Vladimirovne: glavnyj arxitektor provodil restavraciju central'nyx rajonov Volgograda.

7.12.a. В мире художественной гимнастики все знают Ирину Александровну: старший тренер вырастил поколение талантливых спортсменок.

7.12.b. V mire xudožestvennoj gimnastiki vse znajut Irinu Aleksandrovnu: staršij trener vyrastil pokolenie talantlivyx sportsmenok.

7.13.a. У меня не получилось вчера попасть на прием к Ивановой: зубной врач принимал пациентов только по предварительной записи.

7.13.b. U menja ne polučilos' včera popast' na priem k Ivanovoj: zubnoj vrač prinimal pacientov tol'ko po predvaritel'noj zapisi.

7.14.a. Инне Аркадьевне известны все особенности этой стройки: горный инженер разрабатывал вентиляцию для этой шахты год назад.

7.14.b. Inne Arkad'evne izvestny vse osobennosti jetoj strojki: gornyj inžener razrabatyval ventiljaciju dlja jetoj šaxty god nazad.

7.15.a. У Евгении Львовны большой опыт в общении с иностранцами: учёный секретарь организовывал конференцию по индоиранским языкам в прошлом году.

7.15.b. U Evgenii L'vovny bol'šoj opyt v obščenii s inostrancami: učjonyj sekretar' organizovyval konferenciju po indoiranskim jazykam v prošlom godu.

7.16.a. В нашем отделе много учеников Ольги Петровны: системный программист руководил стажерами компании многие годы.

7.16.b. V našem otdele mnogo učenikov Ol'gi Petrovny: sistemnyj programmist rukovodil stažerami kompanii mnogie gody.

8.1.a. Всю ночь Тане не удалось сомкнуть глаз: менеджер готовил презентацию рекламной кампании для радиохолдинга.

8.1.b. Vsju noč' Tane ne udalos' somknut' glaz: menedžer gotovil prezentaciju reklamnoj kampanii dlja radioxoldinga.

8.2.a. Сегодня надо поздравить Анну Михайловну: агент получил премию за успешную продажу полисов.

8.2.b. Segodnja nado pozdravit' Annu Mixajlovnu: agent polučil premiju za uspešnuju prodažu polisov.

8.3.a. Вчера Ивановой вручили премию: режиссёр представил спектакль на престижном фестивале.

8.3.b. Včera Ivanovoj vručili premiju: režissjor predstavil spektakl' na prestižnom festival.

8.4.a. Победой на выборах мы обязаны Ирине: технолог продвигал кандидата весьма успешно.

8.4.b. Pobedoj na vyborax my objazany Irine: texnolog prodvigal kandidata ves'ma uspešno.

8.5.a. У Елизаветы Геннадьевны однозначно кулинарный талант: старший повар подготовил меню для важного банкета за сутки.

8.5.b. U Elizavety Gennad'evny odnoznačno kulinarnyj talant: staršij povar podgotovil menju dlja važnogo banketa za sutki.

8.6.a. Процветанием компании мы обязаны Анне Викторовне: директор обеспечивал участие в крупнейших тендерах города.

8.6.b. Procvetaniem kompanii my objazany Anne Viktorovne: direktor obespečival učastie v krupnejšix tenderax goroda.

8.7.a. Я обратилась за помощью к Виктории Павловне: руководитель подобрал артистов на проект в течение недели.

8.7.b. Ja obratilas' za pomošč'ju k Viktorii Pavlovne: rukovoditel' podobral artistov na proekt v tečenie nedeli.

8.8.a. Слухи о работоспособности Марины преувеличены: дизайнер создавал концепцию парка целый месяц.

8.8.b. Sluxi o rabotosposobnosti Mariny preuveličeny: dizajner sozdaval koncepciju parka celyj mesjac.

8.9.a. Сегодня у Марины состоялся дебют на телевидении: аналитик представил прогноз цен на нефть в передаче «Экономика».

8.9.b. Segodnja u Mariny sostojalsja debjut na televidenii: analitik predstavil prognoz cen na neft' v peredače «Jekonomika».

8.10.a. Как у Ольги Александровны получается все успевать: редактор написал роман о работе в издательстве.

8.10.b. Kak u Ol'gi Aleksandrovny polučaetsja vse uspevat': redaktor napisal roman o rabote v izdatel'stve.

8.11.a. Жители выразили свою признательность Евгении Владимировне: архитектор проводил реставрацию центральных районов Волгограда.

8.11.b. Žiteli vyrazili svoju priznatel'nost' Evgenii Vladimirovne: arxitektor provodil restavraciju central'nyx rajonov Volgograda.

8.12.a. В мире художественной гимнастики все знают Ирину Александровну: тренер вырастил поколение талантливых спортсменок.

8.12.b. V mire xudožestvennoj gimnastiki vse znajut Irinu Aleksandrovnu: trener vyrastil pokolenie talantlivyx sportsmenok.

8.13.a. У меня не получилось вчера попасть на прием к Ивановой: врач принимал пациентов только по предварительной записи.

8.13.b. U menja ne polučilos' včera popast' na priem k Ivanovoj: vrač prinimal pacientov tol'ko po predvaritel'noj zapisi.

8.14.a. Инне Аркадьевне известны все особенности этой стройки: инженер разрабатывал вентиляцию для этой шахты год назад.

8.14.b. Inne Arkad'evne izvestny vse osobennosti jetoj strojki: inžener razrabatyval ventiljaciju dlja jetoj šaxty god nazad.

8.15.a. У Евгении Львовны большой опыт в общении с иностранцами секретарь организовывал конференцию по индоиранским языкам в прошлом году.

8.15.b. U Evgenii L'vovny bol'šoj opyt v obščenii s inostrancami sekretar' organizovyval konferenciju po indoiranskim jazykam v prošlom godu.

8.16.a. В нашем отделе много учеников Ольги Петровны: программист руководил стажерами компании многие годы.

8.16.b. V našem otdele mnogo učenikov Ol'gi Petrovny: programmist rukovodil stažerami kompanii mnogie gody.

4 Paucal construction experiments

The digit stands for lexicalization. “DP”, “PP”, “QP” stand for DP context, PP context, QP context correspondingly. “N” stands for modified feminine nouns, “n_adj” stands for nominalized adjectives. “Anim” and “inanim” stand for animate and inanimate correspondingly.

1.DP.n_adj.anim.a. Две беременные/беременных обсуждали новости сидя на скамейке.

1.DP.n_adj.anim.b. Dve beremennye/beremennyx obsuždali novosti sidja na skamejke.

2.DP.n_adj.anim.a. Две горничные/горничных убирали номер к приезду гостя.

2.DP.n_adj.anim.b. Dve gorničnye/gorničnyx ubirali nomer k priezdu gostja.

3.DP.n_adj.anim.a. Три дежурные/дежурных работали в общежитии во время каникул.

3.DP.n_adj.anim.b. Tri dežurnye/dežurnyx rabotali v obščežitii vo vremja kanikul.

4.DP.n_adj.anim.a. Две знакомые/знакомых подписали открытку ко Дню рождения.

4.DP.n_adj.anim.b. Dve znakomye/znakomyx podpisali otkrytku ko Dnju roždenija.

5.DP.n_adj.anim.a. Три пострадавшие/пострадавших написали заявление о пропаже телефонов.

5.DP.n_adj.anim.b. Tri postradavšie/postradavšix napisali zajavlenie o propaže telefonov.

6.DP.n_adj.anim.a. Три служащие/служащих подготовили обращение на имя президента.

6.DP.n_adj.anim.b. Tri služaščie/služaščix podgotovili obraščenie na imja prezidenta.

7.DP.n_adj.inanim.a. Две прачечные/прачечных были отремонтированы в городе в этом месяце.

7.DP.n_adj.inanim.b. Dve pračečnye/pračečnyx byli otremontirovany v gorode v jetom mesjace.

8.DP.n_adj.inanim.a. Две мастерские/мастерских были оборудованы для сварочных работ.

8.DP.n_adj.inanim.b. Dve masterskie/masterskix byli oborudovany dlja svaročnyx rabot.

9.DP.n_adj.inanim.a. Две шашлычные/шашлычных были освобождены для участников симпозиума.

9.DP.n_adj.inanim.b. Dve šašlyčnye/šašlyčnyx byli osvoboždeny dlja učastnikov simpoziuma.

10.DP.n_adj.inanim.a. Две закусочные/закусочных были открыты в рамках фестиваля.

10.DP.n_adj.inanim.b. Dve zakusočnye/zakusočnyx byli otkryty v ramkax festivalja.

11.DP.n_adj.inanim.a. Три запятые/запятых были поставлены зеленой ручкой.

11.DP.n_adj.inanim.b. Tri zapjatye/zapjatyx byli postavleny zelenoj ručkoj.

12.DP.n_adj.inanim.a. Три гостиные/гостиных были отреставрированы в старинном особняке.

12.DP.n_adj.inanim.b. Tri gostinye/gostinyx byli otrestavrirovany v starinnom osobnjake.

13.DP.n.anim.a. Три веселые/весёлых девочки обсуждали планы на выходные.

13.DP.n.anim.b. Tri veselye/vesjolyx devočki obsuždali plany na vyxodnye.

14.DP.n.anim.a. Две милые/милых подружки играли в песочнице весь день.

14.DP.n.anim.b. Dve milye/milyx podružki igrali v pesočnice ves' den'.

15.DP.n.anim.a. Две греческие/греческих богини пели песню в начале сценки.

15.DP.n.anim.b. Dve grečeskie/grečeskix bogini peli pesnju v načale scenki.

16.DP.n.anim.a. Три деревенские/деревенских девчушки играли в салочки во дворе.

16.DP.n.anim.b. Tri derevenskie/derevenskix devčuški igrali v saločki vo dvore.

17.DP.n.anim.a. Две прекрасные/прекрасных женщины зашли в ресторан поздно вечером.

17.DP.n.anim.b. Dve prekrasnye/prekrasnyx ženščiny zašli v restoran pozdno večerom.

18.DP.n.anim.a. Три нарядные/нарядных девушки ужинали за дальним столиком.

18.DP.n.anim.b. Tri narjadnye/narjadnyx devuški užinali za dal'nim stolikom.

19.DP.n.inanim.a. Две сочные/сочных груши были оставлены в новой вазе.

19.DP.n.inanim.b. Dve sočnye/sočnyx gruši byli ostavleny v novoj vaze.

20.DP.n.inanim.a. Две легковые/легковых машины были припаркованы ночью рядом с домом.

20.DP.n.inanim.b. Dve legkovye/legkovyx mašiny byli priparkovany noč'ju rjadom s domom.

21.DP.n.inanim.a. Три дорогие/дорогих сумки были помещены в витрину магазина.

21.DP.n.inanim.b. Tri dorogie/dorogix sumki byli pomeščeny v vitrinu magazina.

22.DP.n.inanim.a. Три ажурные/ажурных салфетки были оставлены на праздничном столе.

22.DP.n.inanim.b. Tri ažurnye/ažurnyx salfetki byli ostavleny na prazdničnom stole.

23.DP.n.inanim.a. Три грубые/грубых ошибки были помечены красной ручкой.

23.DP.n.inanim.b. Tri grubye/grubyx ošibki byli pomečeny krasnoj ručkoj.

24.DP.n.inanim.a. Две сельские/сельских дороги были пропущены в новом издании атласа.

24.DP.n.inanim.b. Dve sel'skie/sel'skix dorogi byli propuščeny v novom izdanii atlasa.

1.PP.n_adj.anim.a. За каждый час врач принимает по две беременные/беременных.

1.PP.n_adj.anim.b. Za každyj čas vrač prinimaet po dve beremennye/beremennyx.

2.PP.n_adj.anim.a. В каждый зал менеджер вызвал по две горничные/горничных.

2.PP.n_adj.anim.b. V každyj zal menedžer vyzval po dve gorničnye/gorničnyx.

3.PP.n_adj.anim.a. На каждый этаж директор пригласил по три дежурные/дежурных.

3.PP.n_adj.anim.b. Na každyj jetaž direktor priglasil po tri dežurnye/dežurnyx.

4.PP.n_adj.anim.a. На бал каждая выпускница пригласила по две знакомые/знакомых.

4.PP.n_adj.anim.b. Na bal každaja vypusknica priglasila po dve znakomye/znakomyx.

5.PP.n_adj.anim.a. После аварии каждый психолог принял по три пострадавшие/пострадавших.

5.PP.n_adj.anim.b. Posle avarii každyj psixolog prinjal po tri postradavšie/postradavšix.

6.PP.n_adj.anim.a. К каждому гостю руководитель прикрепил по три служащие/служащих.

6.PP.n_adj.anim.b. K každomu gostju rukovoditel' prikrepil po tri služaščie/služaščix.

7.PP.n_adj.inanim.a. В каждом районе компания открыла по две прачечные/прачечных.

7.PP.n_adj.inanim.b. V každom rajone kompanija otkryla po dve pračečnye/pračečnyx.

8.PP.n_adj.inanim.a. Каждую декаду предприятие берет в аренду две мастерские/мастерских.

8.PP.n_adj.inanim.b. Každuju dekadu predprijatie beret v arendu dve masterskie/masterskix.

9.PP.n_adj.inanim.a. Каждое лето предприниматель открывает по две шашлычные/шашлычных.

9.PP.n_adj.inanim.b. Každoe leto predprinimatel' otkryvaet po dve šašlyčnye/šašlyčnyx.

10.PP.n_adj.inanim.a. На страницу путеводителя редактор поместил по две закусочные/закусочных.

10.PP.n_adj.inanim.b. Na stranicu putevoditelja redaktor pomestil po dve zakusočnye/zakusočnyx.

11.PP.n_adj.inanim.a. В этой рукописи каждый абзац содержит по три запятые/запятых.

11.PP.n_adj.inanim.b. V jetoj rukopisi každyj abzac soderžit po tri zapjatye/zapjatyx.

12.PP.n_adj.inanim.a. В каждом коттедже дизайнер обустраивает по три гостиные/гостиных.

12.PP.n_adj.inanim.b. V každom kottedže dizajner obustraivaet po tri gostinye/gostinyx.

13.PP.n.anim.a. На каждую лавочку режиссер посадил по три веселые/весёлых девочки.

13.PP.n.anim.b. Na každuju lavočku režisser posadil po tri veselye/vesjolyx devočki.

14.PP.n.anim.a. За каждой партой учитель посадил по две милые/милых подружки.

14.PP.n.anim.b. Za každoj partoj učitel' posadil po dve milye/milyx podružki.

15.PP.n.anim.a. У каждой колонны режиссер расположил по две греческие/греческих богини.

15.PP.n.anim.b. U každoj kolonny režisser raspoložil po dve grečeskie/grečeskix bogini.

16.PP.n.anim.a. По обе стороны оператор посадил по три деревенские/деревенских девчушки.

16.PP.n.anim.b. Po obe storony operator posadil po tri derevenskie/derevenskix devčuški.

17.PP.n.anim.a. На сцену конферансье приглашал по две прекрасные/прекрасных женщины.

17.PP.n.anim.b. Na scenu konferans'e priglašal po dve prekrasnye/prekrasnyx ženščiny.

18.PP.n.anim.a. Для вручения премии ведущий звал по три нарядные/нарядных девушки.

18.PP.n.anim.b. Dlja vručenija premii veduščij zval po tri narjadnye/narjadnyx devuški.

19.PP.n.inanim.a. Каждому гостю хозяйка выдала по две сочные/сочных груши.

19.PP.n.inanim.b. Každomu gostju xozjajka vydala po dve sočnye/sočnyx gruši.

20.PP.n.inanim.a. В каждый бокс механик поместил по две легковые/легковых машины.

20.PP.n.inanim.b. V každyj boks mexanik pomestil po dve legkovye/legkovyx mašiny.

21.PP.n.inanim.a. На каждую полку продавец положил по три дорогие/дорогих сумки.

21.PP.n.inanim.b. Na každuju polku prodavec položil po tri dorogie/dorogix sumki.

22.PP.n.inanim.a. Каждому гостю хозяйка дала по три ажурные/ажурных салфетки.

22.PP.n.inanim.b. Každomu gostju xozjajka dala po tri ažurnye/ažurnyx salfetki.

23.PP.n.inanim.a. В каждом предложении Петя сделал по три грубые/грубых ошибки.

23.PP.n.inanim.b. V každom predloženii Petja sdelal po tri grubye/grubyx ošibki.

24.PP.n.inanim.a. К каждому поселку компания проложила по две сельские/сельских дороги.

24.PP.n.inanim.b. K každomu poselku kompanija proložila po dve sel'skie/sel'skix dorogi.

1.QP.n_adj.anim.a. Вчера за этот срок принято две беременные/беременных.

1.QP.n_adj.anim.b. Včera za jetot srok prinjato dve beremennye/beremennyx.

2.QP.n_adj.anim.a. Для уборки люкса вызвано две горничные/горничных.

2.QP.n_adj.anim.b. Dlja uborki ljuksa vyzvano dve gorničnye/gorničnyx.

3.QP.n_adj.anim.a. На собрание комитета пришло три дежурные/дежурных.

3.QP.n_adj.anim.b. Na sobranie komiteta prišlo tri dežurnye/dežurnyx.

4.QP.n_adj.anim.a. На фестивале ко мне подошло две знакомые/знакомых.

4.QP.n_adj.anim.b. Na festivale ko mne podošlo dve znakomye/znakomyx.

5.QP.n_adj.anim.a. На заседание суда пришло три пострадавшие/пострадавших.

5.QP.n_adj.anim.b. Na zasedanie suda prišlo tri postradavšie/postradavšix.

6.QP.n_adj.anim.a. В передачу на интервью приглашено три служащие/служащих.

6.QP.n_adj.anim.b. V peredaču na interv'ju priglašeno tri služaščie/služaščix.

7.QP.n_adj.inanim.a. В этом районе за год обустроено две прачечные/прачечных.

7.QP.n_adj.inanim.b. V jetom rajone za god obustroeno dve pračečnye/pračečnyx.

8.QP.n_adj.inanim.a. Для воплощения проекта построено две мастерские/мастерских.

8.QP.n_adj.inanim.b. Dlja voploščenija proekta postroeno dve masterskie/masterskix.

9.QP.n_adj.inanim.a. Около футбольного стадиона оборудовано две шашлычные/шашлычных.

9.QP.n_adj.inanim.b. Okolo futbol'nogo stadiona oborudovano dve šašlyčnye/šašlyčnyx.

10.QP.n_adj.inanim.a. В вашем районе открыто две закусочные/закусочных.

10.QP.n_adj.inanim.b. V vašem rajone otkryto dve zakusočnye/zakusočnyx.

11.QP.n_adj.inanim.a. В эту версию рукописи добавлено три запятые/запятых.

11.QP.n_adj.inanim.b. V jetu versiju rukopisi dobavleno tri zapjatye/zapjatyx.

12.QP.n_adj.inanim.a. В старинном особняке отреставрировано три гостиные/гостиных.

12.QP.n_adj.inanim.b. V starinnom osobnjake otrestavrirovano tri gostinye/gostinyx.

13.QP.n.anim.a. На этот этап викторины прошло три веселые/весёлых девочки.

13.QP.n.anim.b. Na jetot jetap viktoriny prošlo tri veselye/vesjolyx devočki.

14.QP.n.anim.a. К юной гимнастке подбежало две милые/милых подружки.

14.QP.n.anim.b. K junoj gimnastke podbežalo dve milye/milyx podružki.

15.QP.n.anim.a. На древней гравюре нарисовано две греческие/греческих богини.

15.QP.n.anim.b. Na drevnej gravjure narisovano dve grečeskie/grečeskix bogini.

16.QP.n.anim.a. К пожилому фельдшеру подбежало три деревенские/деревенских девчушки.

16.QP.n.anim.b. K požilomu fel'dšeru podbežalo tri derevenskie/derevenskix devčuški.

17.QP.n.anim.a. На афише спектакля нарисовано две прекрасные/прекрасных женщины.

17.QP.n.anim.b. Na afiše spektaklja narisovano dve prekrasnye/prekrasnyx ženščiny.

18.QP.n.anim.a. За дальним столиком замечено три нарядные/нарядных девушки.

18.QP.n.anim.b. Za dal'nim stolikom zamečeno tri narjadnye/narjadnyx devuški.

19.QP.n.inanim.a. На столе к вечеру осталось две сочные/сочных груши.

19.QP.n.inanim.b. Na stole k večeru ostalos' dve sočnye/sočnyx gruši.

20.QP.n.inanim.a. В ремонтном боксе стояло две легковые/легковых машины.

20.QP.n.inanim.b. V remontnom bokse stojalo dve legkovye/legkovyx mašiny.

21.QP.n.inanim.a. На одной из витрин лежало три дорогие/дорогих сумки.

21.QP.n.inanim.b. Na odnoj iz vitrin ležalo tri dorogie/dorogix sumki.

22.QP.n.inanim.a. На праздничном столе осталось три ажурные/ажурных салфетки.

22.QP.n.inanim.b. Na prazdničnom stole ostalos' tri ažurnye/ažurnyx salfetki.

23.QP.n.inanim.a. В контрольной работе отмечено три грубые/грубых ошибки.

23.QP.n.inanim.b. V kontrol'noj rabote otmečeno tri grubye/grubyx ošibki.

24.QP.n.inanim.a. В новом издании атласа пропущено две сельские/сельских дороги.

24.QP.n.inanim.b. V novom izdanii atlasa propuščeno dve sel'skie/sel'skix dorogi.
